# 37 kDa LRP::FLAG enhances telomerase activity and reduces ageing markers in vivo

**DOI:** 10.1007/s00018-025-05593-0

**Published:** 2025-02-22

**Authors:** Tyrone C. Otgaar, Martin Bernert, Gavin Morris, Pavan Baichan, Monique J. Bignoux, Boitelo Letsolo, Stefan F. T. Weiss, Eloise Ferreira

**Affiliations:** School of Molecular and Cell Biology, University of the Witwatersrand, Private Bag 3, Wits, Johannesburg, 2050 Republic of South Africa

**Keywords:** Ageing, LRP, Telomerase, Mitochondrial dysfunction, C57BL/6J mice, Senescence

## Abstract

Ageing is a degenerative process characterised by a decline in physiological functioning of the organism. One of the core regulators of cellular ageing are telomeres, repetitive DNA sequences of TTAGGG that cap the ends of chromosomes and are maintained by the ribonucleoprotein complex, telomerase. Age-dependent progressive loss of the telomere ends eventually induces cell cycle arrest for the induction of either replicative senescence or apoptosis. It was previously established that overexpression of the 37 kDa/ 67 kDa laminin receptor (LRP/LR) increased telomerase activity and telomere length while concomitantly reducing senescence markers in aged normal cells in vitro. Therefore, it was hypothesized that elevating LRP/LR in vivo may increase telomerase activity and hinder the ageing process on an organism scale. To this end, aged C57BL/6J mice were treated/transfected to induce an overexpression of LRP::FLAG. Various physiological tests and histological analyses were performed to assess overall organism fitness as well as to discern the treatments’ ability at reducing tissue degeneration and atrophy. It was found that mice overexpressing LRP::FLAG displayed improved physiological characteristics and markedly less tissue degeneration and atrophy when compared to control and non-treated mice. Alongside these improvements, certain organs displayed increased telomerase activity with a corresponding elongation in average telomere length. In addition the overexpression of LRP::FLAG significantly improved various proliferative and anti-ageing associated proteins while causing a concomitant decrease in senescence associated proteins. These findings are therefore indicative of a novel function of LRP/LR delaying the onset of senescence, while also promoting healthier ageing through elevating TERT and telomerase activity.

**Supplementary Information:**

The online version contains supplementary material available at 10.1007/s00018-025-05593-0.

## Introduction

The 37 kDa Laminin Receptor Precursor/67 kDa High affinity Laminin Receptor (LRP/LR), also known as ribosomal protein SA (RPSA), serves as a cell surface receptor, and as a vital component of the 40S ribosomal subunit [1–3]. The protein has been mapped to the cell surface of the plasma membrane residing within the lipid rafts [4–6], various cytoplasmic regions [7–9] and within the nuclear compartments [8, 10–12], illustrating the protein’s importance by its broad-spanning localisation. LRP/LR carries out major functions on the cell surface as a non-intergrin transmembrane receptor of laminin-1 [2, 3]. Additionally, LRP has been associated with the telomerase reverse transcriptase (TERT) component of the telomerase enzyme to promote telomerase activity for sustained cell viability and prevention of senescence [7, 11, 13, 14]. This functional relationship between LRP/LR and TERT involves cellular survival and viability functions, however the molecular mechanisms underlying these functions are still not entirely understood.

Telomeric erosion is a reduction of telomere length after rounds of cell replication [15–17] and is linked to normal ageing in humans [15, 18–21]. This erosion continues until the “Haylick limit” is reached; a critical length of the telomeres that signals for senescence [15, 22–24]. This form of senescence can be prevented in a controlled manner, through the telomere replenishment actions of telomerase, subsequently leading to an impediment of cellular ageing and increased proliferative capacity [25–30]. The active enzyme is composed of two core sub-units, TERC the RNA subunit that contains the template for reverse transcription, which is read by the catalytic subunit TERT, for the generation of new TTAGGG repeats [31, 32].

Intriguingly, like TERT, LRP/LR appears to be expressed at lower levels in ageing tissue, and we have previously shown that LRP::FLAG overexpression significantly elevated hTERT levels, telomerase activity and telomere length in aged cells. Concomitantly, LRP::FLAG overexpression reduced the levels of the senescence/ageing markers β-galactosidase and γH2AX in both HEK293 and MRC-5 cells [14]. In relation, LRP::FLAG overexpression rescued HEK293 and SH-SY5Y cells from Aβ-mediated cytotoxicity, improved overall cell viability, and enhanced telomerase activity while in the presence of cytotoxic levels of Aβ [7]. Collectively, these findings support the theory that a functional relationship exists between LRP and TERT, to ultimately promote proliferation and viability in both normal and pathological settings. We therefore investigated the effect of LRP::FLAG overexpression on various physiological and cognitive characteristics, tissue structure, telomerase activity, telomere lengthas well as gene and protein expression profiles of various senescence, proliferative and anti-senescence markers in aged mice to determine if LRP/LR was capable of modulating the senescence process.

## Methodology

### Animal trials and treatments

The study involved the use of female C57BL/6J mice at approximately 12 months of age (Charles River Laboratories, France). Mice were housed in separate cages (plexiglass, 28 × 12 × 16 cm) with sawdust for bedding and provided with free intake of food and water, 22 ± 2 ℃, humidity 50–60% and a 12 h light: dark cycle. Forty mice were acquired and 36 of these mice were randomized into the 3 treatment groups. These groups included group 1, which received the pCIneo-moLRP::FLAG plasmid and TurboFect™ in vivo Transfection Reagent (Thermo Fisher Scientific, Massachusetts, USA) to induce LRP::FLAG overexpression. The use of a cationic transfection reagent was employed as it has been previously shown to enhance plasmid uptake in vivo in several studies and animal models [33–37]. Group 2 received the empty pCIneo plasmid and transfection reagent (vector control). Lastly, group 3 received a 5% glucose solution that served as a vehicle control. Prior to treating the 3 different mouse groups a pilot study was conducted on three non-sorted mice to assess treatment efficacy. This was carried out over a 1 month period and therafter the mice in the three treatment groups were allowed to age for a further month before the full study commenced (mice age ± 15 months). The treatment involved the use of two administration routes: a tail vein injection and an intramuscular injection with treatments spaced 10 days apart. In fact, previous studies have shown that use of a cationic polymer for plasmid delivery by intravenous injection allow plasmid delivery predominantly to the lungs and to a lesser extent the kidney, liver and spleen [33, 34, 36]. Additionally, intramuscular injections of naked or encapsulated plasmid allows for muscle transfection and plasmid expression [38, 39]. Thereafter, the treatment was repeated ± 5 months after the initial treatment to ensure a widespread delivery of the plasmid to various tissue sites. This strategy was selected as a transient transfection was assumed as previous studies have shown that plasmid persistence differs between tissue types with the plasmid generally being detectable only for periods of 3–30 days [33, 39]. All treatments were performed according to the manufacturer’s recommendations. Briefly, the tail vein injection involved diluting 50 µg of purified plasmid DNA (either pCIneo-moLRP::FLAG construct or pCIneo plasmid) in 400 µl of sterile 5% glucose solution with the addition of 6 µl of the TurboFect™ (Thermo Scientific) in vivo Transfection Reagent. After a 15–20 min incubation at RT each mouse received 400 µl treatment via tail vein injection. The intramuscular injection involved diluting 85 µg of purified plasmid to a volume of 96 µl in sterile 5% glucose with the addition of 4 µl of TurboFect™. Following, a 15 min incubation at RT, each mouse received 100 µl of the plasmid solution or 5% glucose (vehicle control) by intramuscular injection into the right and left quadriceps (50 µl per injection). Physiological tests were carried out ± 6 weeks after the first treatment. The second round of treatment was performed 10 days prior to euthanization. During the course of the study the following mice had to be prematurely terminated: Treated mice: 1 mouse due to the development of cancer (enlarged spleen with evidence of growths); Control mice: 1 mouse due to a persistent anal prolapse, 1 mouse groomed excessively which led to self-harm and also experienced weight loss overtime, 3 mice died during the course of the second treatment from anaemia, a common condition in senior mammals; Non treated: 1 mouse due to a persistent rectal prolapse. After all the mice were euthanised and tissue sampling took place the tissue and organs were inspected for growths and tissue abnormalities. Aside from the mice that were prematurely terminated a further 2 mice (1 treated and 1 non-treated due to the formation of growths and enlargement of the liver (treated) and spleen (non-treated)) were removed and not utilised for biochemical analysis. In addition, a study flow chart (Fig. S1a, b) has been included that details when procedures were performed and at what approximate age and weight the mice were at the time of the procedures. All experimental procedures were performed with approval by the University of the Witwatersrand Animal Ethics Screening Committee (clearance certificate number: 2016/08/38/B).

### Hair greying analysis

All mice were imaged prior to termination using a Canon E05 450D. The mice were imaged in a brightly lit room on a neutral green background to minimise light and colour interferance. The images were analysed using Gandalf the Grey, an in-house software developed by Dr G. Morris. The software measured the amount of grey and white pixels present on a mouse’s coat. This data was then presented as a percentage compared to the total amount of black pixels present on the coat.

### Motor function analysis using the balance beam test

The procedure involved using 1 m long beams with a width of 12 mm that were suspended between two poles; approximately 50 cm above a cushioned surface. The mouse was then placed at one end of the beam while the other end housed a platform and goal box containing nesting material from the home cage as well as food [40]. Thereafter, the mouse was allowed to traverse the beam. The tests were recorded using a Canon E05 450D where the time it took each mouse to traverse the beam as well as the approximate number of foot-slips were analysed [40]. In addition, each mouse was also scored on its ability to traverse the beams; scores were allocated as follows: (1) Mouse cannot traverse the beam, (2) Mouse traverses the beam while dragging its hindlimbs, (3) Mouse traverses the beam with some dragging followed with attempted use of hindlimbs with slips, (4) Mouse traverses the beam using all four limbs with a large number of slips (> 20), (5) Mouse traverses the beam using all four limbs with a small number of slips (< 20), (6) Mouse traverses the beam using all four limbs without slipping.

### Puzzle box test

The protocol adapted from Ben Abdallah et al. (2011) [41] was utilised with slight modifications incorporated. The testing arena consisted of a white container separated by a detachable barrier into two compartments: an empty brightly lit start zone (67 cm long, 25.5 cm wide) and a smaller dark and covered goal zone (15 cm long, 25.5 cm wide) filled with sawdust. Each mouse was individually placed into the testing arena in the start zone and was allowed to travel into the goal zone by entering a narrow underpass (± 4 cm) set into the barrier. Each mouse was then subjected to a total of 9 trials (T1- T9) of increased complexity over a 3 day period. On Day 1 (T1) the underpass was un-obstructed and the top was uncovered. During T2 and T3 the top of the underpass was covered. On Day 2, T4 was a repetition of T2 and T3. Thereafter, T5 and T6 (burrowing puzzle) the underpass was filled with sawdust, which the mouse had to burrow through to locate the goal zone. On Day 3, T7 was a repetition of T5 and T6. In regard to T8 and T9, a more complex plug and burrow puzzle was introduced, where the underpass was obstructed with rolled up gauze that was overlaid with sawdust. This required the mouse to burrow as well as to pull or push the gauze out in order to enter the goal zone. The setup of T1- T9 in this manner allowed the assessment of problem solving ability (T5 and T8), learning and short term memory (T3, T6 and T9), while the repetition performed the following day evaluated long term memory (T4 and T7). Each mouse was assessed based on its performance by measuring the time taken to enter the goal zone with all 4 paws or the trial was ended after a period of 3 min. All tests were video recorded using a Canon E05 450D.

### Novel object recognition

The novel object recognition (NOR) test was performed in a 440 mm × 500 mm open field box with opaque walls. Each mouse was habituated to an empty open field box for 10 min, prior to conducting the NOR and social interaction tests. Each mouse was subjected to a 5 min training session of exposure to two identical, non-toxic, hard plastic items in the open field box. After the training session, the mouse was returned to its home cage. After 2 h (testing short term memory), the mouse was returned to the arena which contained two objects, one identical to the familiar object and one novel object. The animal was allowed to explore for 5 min, during which the amount of time exploring each object was recorded and tracked. This was repeated after 24 h (testing long term memory) where the mouse was returned to the arena which contained two objects, one identical to the familiar object and one novel object (different to the novel object from the previous day). ToxTrac software (Rodriguez, A, Zhang, H, Klaminder, J, Brodin, T, Andersson, PL, Andersson, M. ToxTrac: A fast and robust software for tracking organisms. Methods Ecol Evol. 2018; 9: 460– 464. 10.1111/2041-210X.12874) was used to analyse the time spent exploring the known and novel objects.

### Social interaction

The social interaction test, which assessed behaviour towards an unknown mouse, was performed in a 440 mm × 500 mm open field box with opaque walls. Each mouse was habituated to the empty open field box for 10 min, prior to conducting the NOR and social interaction tests. The unknown mouse was placed into the open field box inside a plastic cage and the test mouse was then placed in the corner of the open field box containing the cage and a non-toxic, hard plastic item. The test mouse’s interactions were then video recorded for a 10 min period using a Canon E05 450D. Thereafter, the videos were analysed using ToxTrac software to measure the percentage amount of time the test mouse spent interacting with the caged unknown mouse and the plastic object.

### Nesting test

Nest building is a common behaviour in mice (confirmed in the C57BL/6J mouse strain) that has been shown to become impaired during cognitive dysfunction or decline that commonly occurs during ageing [42]. The test was performed in the “home” cage of the mouse, where all old nesting material was removed and a single sheet of paper towel was placed into each cage in the same location and left overnight. Thereafter, the nests were imaged and graded according to a 6 point scoring system: (1) Paper towel was not noticeably touched or manipulated, (2) Paper towel exhibited slight folding with no tearing or flat paper towel with minimal tearing, (3) Partial folding and minimal tearing of paper towel, (4) Identifiable nest, with partial folding and shredded material, (5) Nest with well-developed walls, with a distinct amount of folding and/or shredding, (6) Nest in the shape of a cocoon with partial or complete roof. All nests were imaged with a Canon E05 450D for analysis.

### Organ harvest

After all the physiological tests and the second round of treatments had been completed, mice were euthanized and transcardially perfused with cold 1X PBS (Sigma-Aldrich). Mice were approximately 19 months of age at the time of euthanization and the number of mice terminated per group included: 10 mice from the treated group, 7 mice from the vector control group and 11 mice from the vechile control group (Non-treated). The brain, kidney and liver were removed and each sectioned into 2 portions. The first portion was snap-frozen in liquid nitrogen and stored at − 80 °C until used for biochemical analysis. The remaining portions were fixed in 4% paraformaldehyde (PFA) for histological analysis. Briefly, the organs were fixed for 24 h at 4 °C in 4% PFA after which they were rinsed with 1X PBS. Thereafter, the organs were placed in a 30% sucrose solution and incubated for 3 days to displace all water from the tissue. Samples were then stored in 4% PFA to prevent contamination until further analysis was performed.

### Histological analysis

Histological sectioning and staining were performed by Ms. Hasiena Ali, Senior Technician, School of Anatomical Sciences, Faculty of Health Sciences, University of Witwatersrand (Johannesburg, South Africa). All organ samples (brain and kidney) sections were made and processed according to routine histological tissue processing in an automated tissue processor with standard operating procedures. Following tissue processing, sections of 4–5 μm were cut and the produced slides were stained in an automated Haematoxylin and Eosin tissue stainer. The stained slides were then visualised and imaged using the Zeiss Primovert microscope (Zeiss, Oberkochen, Germany), where tissue atrophication and degeneration was assessed. In this regard, analysis of the brain histology incorporated a qualitative approach to assess overall degeneration and the number of cells spanning the width of the dentate gyrus was also analysed to provide a quantitative measurement. In addition, the analysis of the kidney histology involved counting and comparing the number of atrophic/degenerated glomeruli compared to the total number of glomeruli counted to obtain a percentage of atrophic/degenerated glomeruli per mouse that was then compared across treatment groups.

### RNA extraction and qPCR

RNA was extracted from the frozen tissue samples of the brain, kidney and liver for the quantification of various proliferative as well as senescence gene expression levels. The RNA was isolated using the Nucleospin^®^ RNA kit (Macherey-Nagel GmbH & Co. KG, Düren, Germany) by following the manufacturer’s protocol. Thereafter, the isolated RNA was subjected to an additional in-solution DNase treatment utilising the Turbo DNA- free™ kit (Thermo Fisher Scientific, Massachusetts, USA) according to the manufacturer’s recommendations. The Nanodrop One (Thermo Scientific, Massachusetts, USA) was utilised to quantify the purified RNA samples while RNA intergrity and purity were assessed by resolving on a 1% agarose gel (w/v). For qPCR, 1 µg of RNA was used as a template for cDNA synthesis utilising the SensiFAST™ cDNA Synthesis Kit (Bioline, London, UK) adhering to the manufacturers protocol. Quantitative PCR was then performed on the CFX96™ Touch qPCR System (Bio- Rad, California, USA) using the Luna^®^ Universal qPCR Mastermix (New England Biolabs, Massachusetts, USA) with the reaction mix setup as follows: 5 µl of Luna^®^ Universal qPCR Mastermix, 10 µM of each primer, 3 µl of dH_2_O and 50 µg of cDNA sample. In addition, all qPCR reactions were performed alongside appropriate no template and no reverse transcriptase controls. All qPCR reactions were performed under the following cycling parameters: one cycle at 95 °C for 3 min followed by 40 cycles at 95 °C for 10 s and a primer specific temperature (Table S4) for 30 s. To ensure PCR amplification specificity, a melt curve was generated to validate each run. Finally, the relative expression of each gene was calculated by the ∆∆CT method using the Bio-Rad CFX Maestro analysis software.

### Western blotting analysis

Frozen organ tissue samples were homogenized in RIPA buffer and lysed at 4 °C for 30 min with constant agitation. Thereafter, the sample supernatant was collected post centrifugation at 16 000 xg for 20 min and a BCA assay was performed and protein samples were made up to a standardised concentration. The protein samples were then resolved by Sodium Dodecyl Sulphate Polyacrylamide Gel Electrophoresis (SDS- PAGE) using the Mini Protean gel system (Bio- Rad, California, USA) and a 10% polyacrylamide gel. The resolved proteins were then transferred onto a polyvinylidine fluoride (PVDF) membrane (Pall, New York, USA) with 1 × transfer buffer (20% methanol in 25 mM Tris and 19.2 mM glycine) using the Trans-Blot^®^ Turbo™ Transfer system (Bio- Rad, California, USA). The membranes were then blocked in 3% BSA (Glentham Life Sciences, Corsham, UK) in PBS and 0.1% Tween 20 (PBST) for 1 h at RT with gentle agitation. The primary and secondary antibodies as well as their concentrations utilized for western blots are detailed in Table S5. The membranes were visualised by use of the Clarity™ Western ECL Blotting Substrate (Bio- Rad, California, USA) with the ChemiDoc™ Imaging System (Bio- Rad, California, USA) and densitometric analysis was performed with Image Lab 5.1 software (Bio- Rad, Hercules, California, USA). It must be noted that all the samples were fitted across 4 blots with a minimum of three samples per group. In addition, one random technical repeat was performed for each group for blot 2, 3 and 4.

### Assessment of telomerase activity

A modified version of the Telomerase Repeated Amplification protocol was used to determine the effect of LRP::FLAG overexpression on telomerase activity in the various aged mouse organs (brain, kidney and liver). The fractionated homogenates (50 mg) were further homogenized in 200 µl ice cold CHAPS lysis buffer and were then incubated at 4 °C with constant agitation for 30 min. The extracted samples were centrifuged at 16 000 xg for 20 min at 4 °C and the harvested supernatant was subsquently snap frozen on dry ice. The protein samples were then standardised to a concentation of 5 µg/µl using the NanoDrop One (Thermo Fisher Scientific, Massachusetts, USA) and the qPCR reaction was then performed. Briefly, 5 µg/µl of protein samples were analysed alongside a set of TSR8 standards, positive control, heat treated control samples (heat treated at 90 °C for 20 min) and NTC that were mixed with the TRAP reaction mix to make up a total volume of 25 µl. The reaction mix was composed of the following: 100 ng/µl TS primer, 100 ng/µl ACX primer (Table S4), 1 mM EGTA, 0.1 µg/µl ultra-pure BSA, Luna^®^ Universal qPCR Mastermix (New Englxand Biolabs, Massachusetts, USA) and DNase/RNase free H_2_O. The CFX96™ Touch qPCR System (Bio- Rad, California, USA) was used with the following cycling conditions: 37 °C for 1 h to allow the extracted telomerase enzymes to extend the telomeric substrate, 95 °C for 2 min and 45 cycles of 95 °C for 15 s, 59 °C for 60 s and 45 °C for 10 s. Thereafter, telomerase activity was calculated from the TSR8 standard curve and the data was analysed using the Bio-Rad CFX Maestro software.

### Telomere length determination

DNA was extracted from frozen tissue homogenates of the brain, kidney and liver for the quantification of telomere length using the Invisorb^®^ Spin Tissue kit (Invitek Molecular, Berlin, Germany) and followed the manufacturer’s protocol. Thereafter, sample intergrity was confirmed by resolving on a 1% agarose gel and samples were then quantified using the NanoDrop One (Thermo Scientific, Massachusetts, USA). Mean telomere length was assessed by use of the qPCR-based procedure described by Cawthon et al., (2002). All qPCR reactions were performed using the appropriate primers and Sensi-Fast SYBR green master mix [(Bioline, London, UK) (Table S4)]. Acidic ribosomal phosphoprotein (36B4) was utilised as a reference gene and three technical repeats were performed for each organ sample. The procedure was perfomed on the CFX96™ Touch qPCR System (Bio- Rad, California, USA) with the following cycling program: Denaturation at 95 °C for 10 min followed by 45 amplification cycles of: 95 °C for 10 s, 58 °C for 10 s and 72 °C for 60 s. Post amplification, mean telomere length was calculated by use of the [2ΔCT (telomeres)/2ΔCT (36B4)] equation.

### Mitochondrial DNA content determination

Mitochondrial DNA content was measured via qPCR [43]. This procedure was carried out as aged cells readily produce an elevated amount of mitochondrial mass/content, however, are generally dysfunctional and thus act as an efficient marker to track senescence. The procedure involved the analysis of both mtDNA and reference gDNA (Table S4) on the same plate to ensure experimental accuracy. All qPCR reactions were performed using the appropriate primers and Sensi-Fast SYBR green master mix (Bioline, London, UK). In addition, the samples were analysed alongside a set of NTCs for each primer set. Thereafter, samples were amplified on the CFX96™ Touch qPCR System (Bio- Rad, California, USA) with the following cycling parameters: an initial denaturation at 95 °C for 3 min, followed by 40 cycles of denaturation at 95 °C for 10 s and a combined annealing/extension step at 60 °C for 30 s. A melt curve was generated to ensure amplification specificity afterwhich samples were analysed using the ∆∆CT method with the Bio-Rad CFX Maestro analysis software.

### Data and statistical analysis

All experimental data collected was subjected to statistical analysis using Graphpad Prism, Microsoft Excel 365 ProPlus (Microsoft Corporation) and QuickCalcs Outlier Calculator ©2017 that makes use of the Grubbs test for the identification of outlier data points. All experimental procedures were performed with a minimum of 7 biological repeats with error bars representing standard deviation. All physiological tests were performed with Mice overexpressing LRP::FLAG (Treated): *n* = 10; Empty vector treated mice (Control): *n* = 11; Non-treated mice: *n* = 11. For histology, and all biochemical analyses with the exception of mRNA gene expression analysis the following group sample sizes were used: Treated: *n* = 9; Control: *n* = 7; Non-treated: *n* = 10 (except for western blotting where *n* = 9). In the event that the material of interest (protein, DNA or RNA) was of poor quality, degraded or had insufficient yield, the samples were excluded from the analysis set. In addition, all gene expression and mitochondrial DNA content analyses data is expressed as a fold change. In terms of statistical analysis, a One-way ANOVA and Welch’s *t-test* coupled with a *post hoc* Bonferroni correction were performed at a 95% confidence interval; where p values < 0.05 were considered statistically significant (*p < 0.05, **p < 0.01 and ***p < 0.001). All statistical calculations as well as the reported means and standard deviations for all experimental procedures are reported in tables: Table S1-S3.

## Results

### The effect of LRP::FLAG overexpression on physiological characteristics and cognitive abilities

**Fig. 1:**
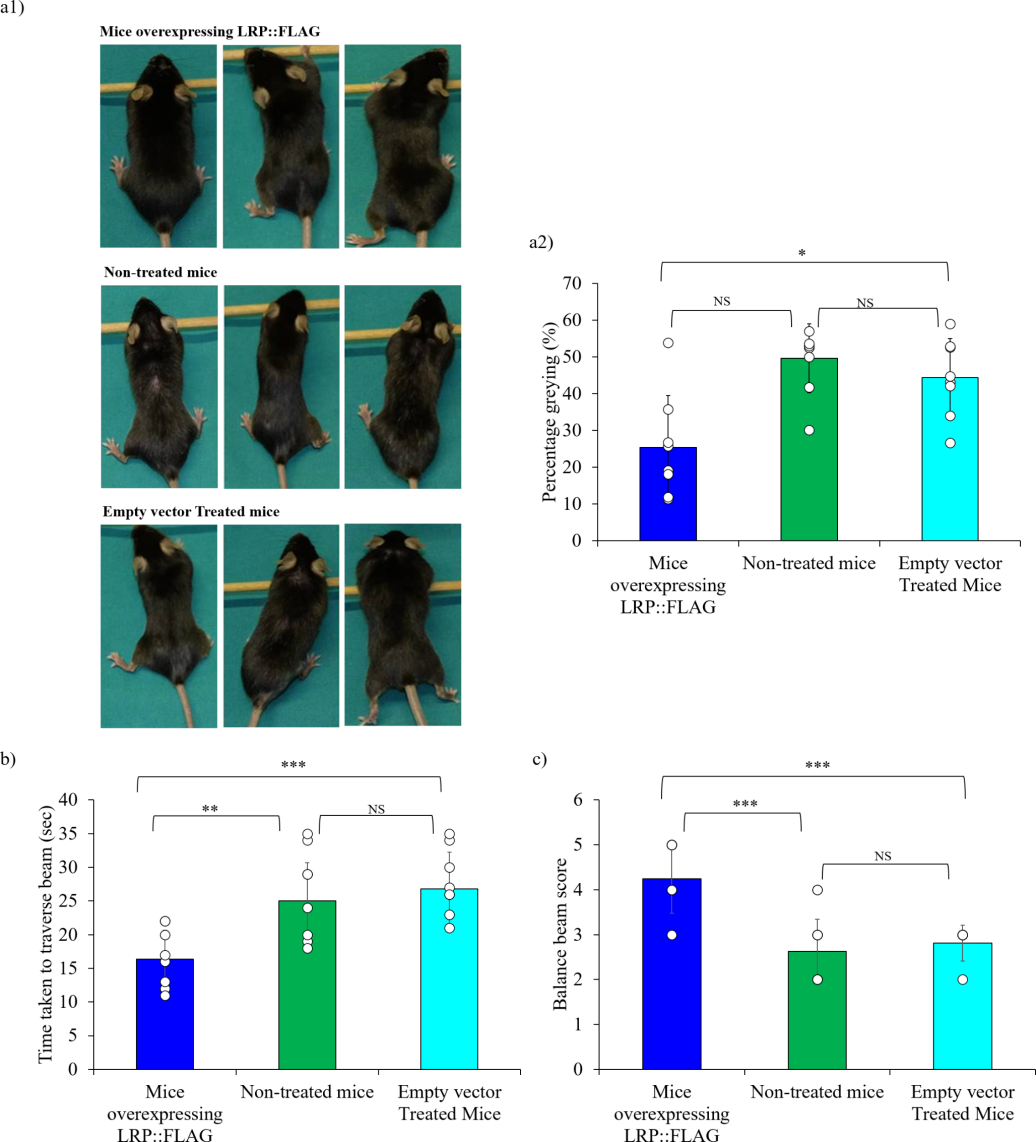
LRP::FLAG overexpression induced a significant improvement in physiological parameters within late adult to old mice. **a**) Hair greying analysis performed on 18 month old mice following LRP::FLAG overexpression. Representative images illustrate treated mice contained less hair greying and less balding. Sample size: Mice overexpressing LRP::FLAG (Treated): *n* = 8; Non-treated mice (Non-treated): *n* = 9 Empty vector treated mice (Control): *n* = 9. **b**) Balance beam test scored the mice’s ability to traverse the beam and **c**) illustrates the time taken to traverse the beam. Data shown is of two averaged technical repeats per biological replicate. Sample size: Treated: *n* = 8; Control: *n* = 8 and Non-treated: *n* = 8. Data represented as the mean ± SD. *p < 0.05, **p < 0.01, ***p < 0.001; One-way ANOVA and Welch’s t-test coupled with Bonferroni correction

We have previously found that LRP::FLAG overexpression in HEK293 and MRC-5 cells significantly reduced ageing markers, while concomitantly improving TERT and telomerase activity levels as well as telomere length. We therefore aimed to confirm this in an in vivo setting using mice, which were treated to overexpress LRP::FLAG. The treatments were completed when the mice were approximately 16 months of age and thereafter physiological tests were conducted when the mice were 17 months of age (late adult to old), while histological and biochemical analysis were performed on tissue from mice that were ± 20 months of age (Fig. S1a). Various physiological tests were conducted to determine LRP’s ability to waylay the effects of physiologic ageing. Firstly, hair greying analysis was performed, as it is one of the most distinguishing phenotypic markers of mammalian ageing and has been confirmed in the C57BL/6J mouse strain. Our analysis revealed that mice treated to overexpress LRP::FLAG (treated mice) displayed a significantly lower percentage (25.38% ±14.11%) of hair greying compared to control mice (empty vector treated mice) (44.42% ±10.58%) and a trend for improvement post Bonferroni correction compared to non-treated (46.68% ±12.59%) mice (Fig. 1a1,2, Table S1). Moreover, it is noteworthy that although not quantified, it was visually observed that the treated mice presented fewer signs of hair thinning and balding, compared to control and non-treated groups.

Thereafter, motor function was assessed by the balance beam test, as a decline is induced by muscle atrophy, a direct consequence of senescent tissue accumulation [44–46]. Importantly to note the average weight of the mice across all three treatment groups (treated: 26.5 g; control: 26.4 g; non-treated: 26.3 g) did not significantly differ and therefore weight would not have affected balance and performance (Fig. S1b). On average, the treated group were able to traverse the 1 m beam in a significantly shorter time (16.375 s ± 4.14 s), compared to both control (26.813 s ± 3.90 s) and non-treated (25 s ± 4.43 s) mice (Fig. 1b, Table S1). Additionally, each mouse was scored on their physical ability to traverse the beam and treated mice displayed a significant improvement in motor function and balance (4.25 ± 0.755) compared to non-treated (2.62 ± 0.72) and control (2.81 ± 0.403) mice (Fig. 1c, Table S1). In fact, treated mice were able to effectively traverse the beam using all four limbs, albeit with slipping, whereas the control and non-treated mice on average resorted to traversing the beam with a large number of slips and by dragging their hindlimbs which could be indicative of an age-dependent decline in muscle mass, motor function and balance.

**Fig. 2:**
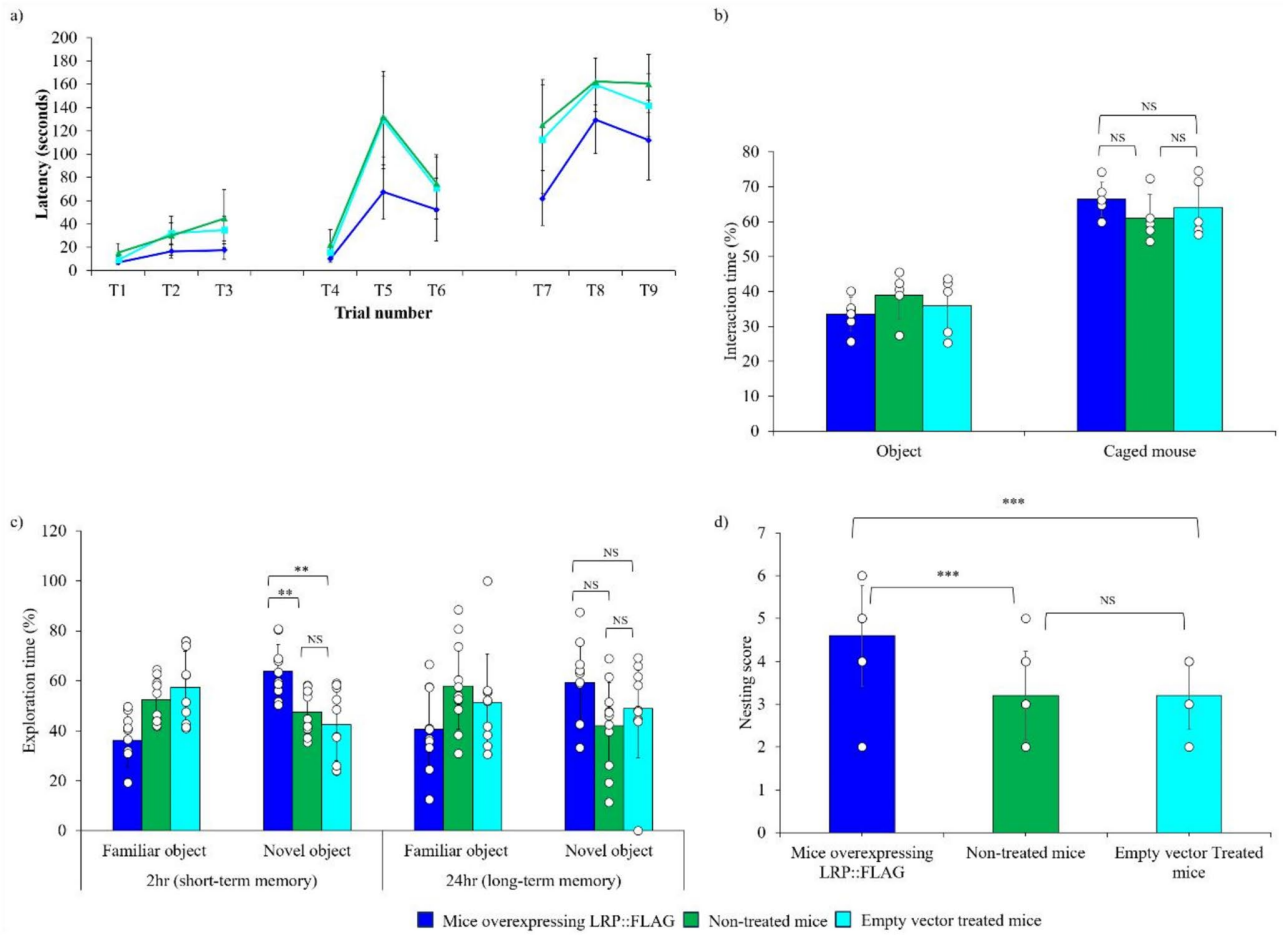
LRP::FLAG overexpression induced a significant improvement in various cognitive parameters within late adult to old mice. **a**) Puzzle box test measured the latency (time) of each mouse to solve cognitive tasks over 9 trials. Day 1 (training), T1 - underpass uncovered and unblocked, T2 and T3 the top was covered, and mice accessed the goal zone via the underpass. Day 2 (burrowing puzzle), T4 was a repeat of T2 and T3. T5 and T6 the underpass was filled with sawdust. Day 3 (plug puzzle), T7, was a repeat of T5 and T6. T8 and T9 the underpass was plugged with gauze and covered with sawdust. Treated: *n* = 10; Control: *n* = 11 and Non-treated: *n* = 11. **b**) Social interaction test measured the total time percentage that each mouse spent interacting with a caged mouse compared to interacting with an object. Sample size: Treated: *n* = 6; Control: *n* = 5 and Non-treated: *n* = 5. **c**) Novel object recognition test measured the total time percentage that each mouse spent exploring a novel object compared to exploring a familiar object. Sample size: Treated: *n* = 10; Control: *n* = 10 and Non-treated: *n* = 10. **d**) Nesting test graded each mouse’s nest-building ability. Sample size: Treated: *n* = 9; Control: *n* = 10 and Non-treated: *n* = 11. Data represented as the mean ± SD. *p < 0.05, **p < 0.01, ***p < 0.001; One-way ANOVA and Welch’s t-test coupled with Bonferroni correction

Cognitive decline is a well-documented characteristic that develops and progresses with increased age in both mice and humans. Cognitive function was assessed using the puzzle box test, which assesses short (T3, T6 and T9) and long-term memory (T4 and T7) as well as puzzle-solving ability (T5 and T8). It was found that treated mice had vastly improved cognitive abilities in all three categories compared to non-treated and control mice (Fig. 2a). In fact, control and non-treated mice exhibited higher latencies in reaching the goal zone for every trial (except T1), compared to treated mice. Therefore, treated mice displayed acute remembrance and enhanced learning capabilities of the opening, burrow and plug puzzle. Social interaction data displayed no difference in interactions of the caged mouse between the different groups, however, mice in all groups displayed a preference for interacting with the caged mouse rather than a novel object illustrating that the treatment had no effect on mouse sociability (Fig. 2b, Table S1). In addition, a significant improvement for short-term memory (Treated (T): 63.81% ±10.77%, Control (C): 42.50% ±14.46%, Non-treated (NT): 47.58% ±8.77%) and a trend for improvement was noted for long term memory (T: 59.38%, C: 48.8%, NT: 42.03%) in the novel object recognition test, where treated mice spent significantly more time exploring the novel objects compared to the known objects (Fig. 2c, Table S1). Furthermore, improved spatial cognitive behaviour was observed in the treated group through improved nest building, which has been confirmed to decline with age in C57BL/6J mouse strain [47]. Treated mice displayed superior nest constructing abilities (4.89 ± 0.782), whereas non-treated (3.2 ± 0.79) and control (3.2 ± 1.03) mice (Fig. 2d, Table S1) displayed limited nesting capabilities.

### LRP::FLAG overexpression delays tissue degeneration

**Fig. 3:**
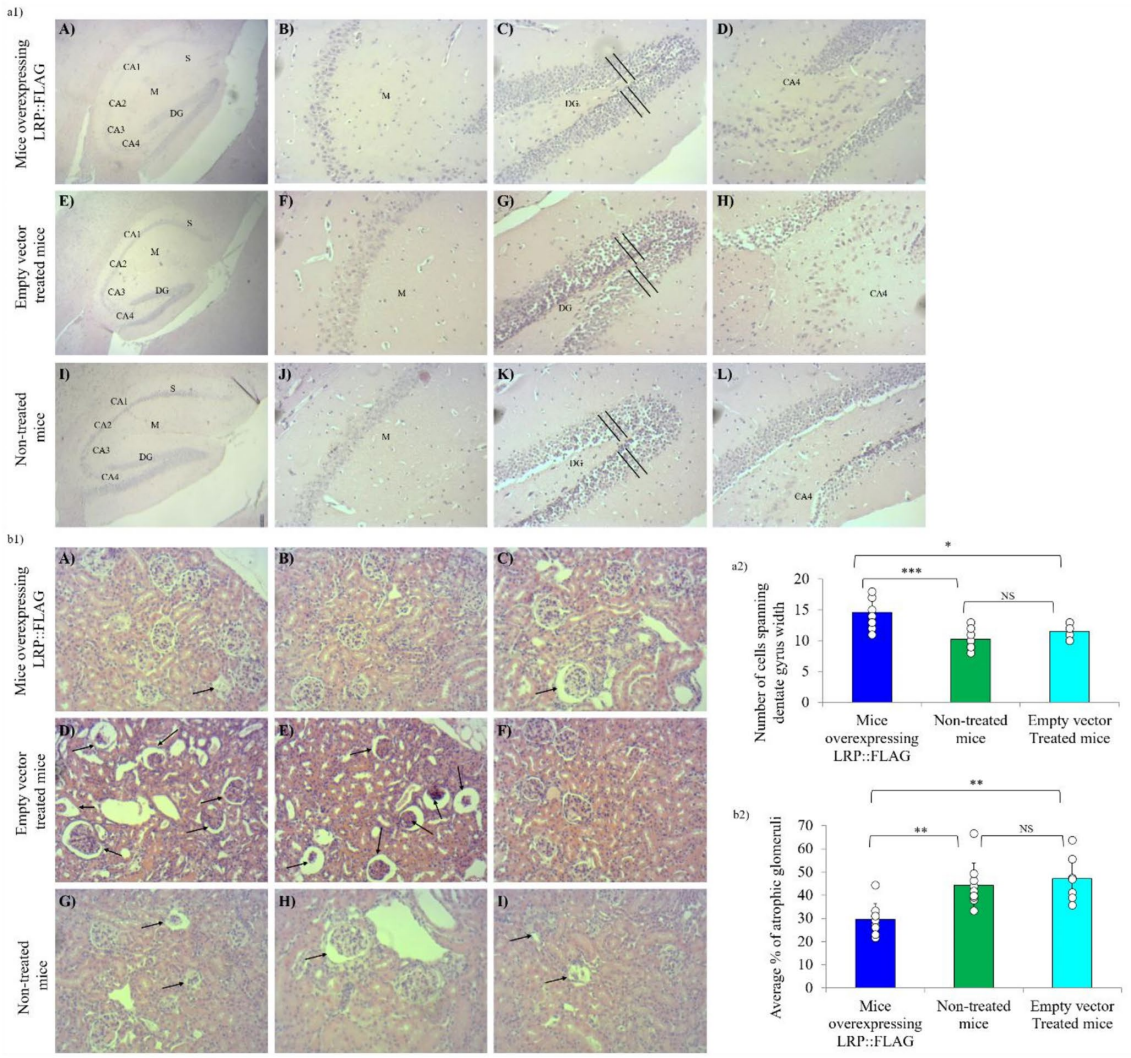
LRP::FLAG overexpression delays the formation of histopathological markers that constitute tissue ageing. **a1**) Histological analysis of the hippocampus following LRP::FLAG overexpression. Brain sections were stained with H&E and images were taken at 40x (**a1**; **A**, **E**, **I**) and 200x (**a1**; **B**-**D**, **F**-**H**, **J**-**L**) magnification. Hippocampus structures assessed: Cornu Amonis 1–4 (CA1-CA4), Dentate gyrus (DG), Subiculum (S) and Molecular layer (M). Evaluation of the molecular layer (**a1**; **B**, **F**, **J**) revealed treated mice on average showed less vacuolation and enlarged neurons. Assessment of the DG (a1; C, G, K) showed treated mice had less signs of tissue vacuolation than the control and non-treated mice. Analysis of the CA (**a1**; **D**, **H**, **L**) indicated control and non-treated mice exhibited more necrotic cells and tissue disorganisation compared to treated mice. **a2**) The average number of cells spanning the width of the DG (marked section) was counted and compared between mouse groups. **b1**,2) Histological analysis of the kidney sections following of LRP::FLAG overexpression. Sections were stained with H&E and imaged at 200x magnification. (**b1**; **A**-**C**) Treated mice on average exhibited normal renal structures with marginal glomerular shrinkage and atrophy. (**b1**; **D**-**F**) Control mice and (**b1**; **G**-**I**) non-treated mice displayed distinct glomerular atrophy and sclerosis (indicated by the arrows) for some biological replicates, while overall showed a larger accumulation of: glomerular shrinkage, vacuolation and inflammatory infiltration (glial cells) than the treated mice. **b2**) For quantification, the average number of counted atrophic glomeruli was offset against the total number of counted glomeruli (healthy and atrophic) per mouse. The average ratios of each group were then compared. Data represented as the mean ± SD. *p < 0.05, **p < 0.01, ***p < 0.001; One-way ANOVA and Welch’s t-test coupled with Bonferroni correction

We subsequently performed histological analysis to assess the kidney and brain. The brain was selected due to the differences in cognitive abilities noted in the treated mice, while the kidney was selected due to noticeable age-related modifications of the glomerular and tubular structures. Moreover, brain tissue analysis focused on the hippocampal region as it serves a vital role in learning and long-term memory [48]. Moreover, the age-dependent functional decline of this neuronal region is directly responsible for the forgetfullness and learning impairments commonly observed in the elderly [48]. Qualitative analysis revealed that while all mice did show mild signs of age-dependent tissue decline, it was more pronounced in non-treated and control mice (Fig. 3a1A-L). On average, treated mice displayed fewer enlarged neurons and less tissue vacuolisation than the non-treated and control groups (Fig. 3a1B, D, F, H, J, L). Furthermore, it was evident that the non-treated and control mouse groups had a less defined dentate gyrus (DG) and a greater number of pyramidal cells containing pyknotic nuclei, compared to the treated mice (Fig. 3a1C, G, K). Treated mice also displayed less vacuolisation, and more microglia with distinct nuclei in the hippocampal region, compared to control and non-treated mice. This was confirmed quantitatively, where on average treated mice had a larger number of cells (14.63 ± 2.56) spanning the width of the DG, compared to non-treated (10.25 ± 1.67) and control (11.5 ± 1.38) mice (Fig. 3a2, Table S1). Kidney analysis revealed that all mice displayed a subset of glomeruli with increased diameters; a commonplace phenomenon that occurs as mice age, although, both untreated and control groups displayed more signs of age-related tissue degeneration compared to treated mice (Fig. 3b1A-I) [49, 50]. Qualitatively, a few signs included: glomerular shrinkage, tubular vacuolation, increased infiltration of lymphocytic (glial) cells throughout the tissue, and signs of mild to severe atrophy. In fact, quantitative analysis revealed that treated mice displayed a lower percentage (29.66% ±6.31%) of atrophic glomeruli compared to both control (47.08% ±9.13%) and non-treated (44.20% ±9.10%) mice (Fig. 3b2, Table S1).

### LRP::FLAG overexpression increases LRP levels and improves telomere dynamics

**Fig. 4:**
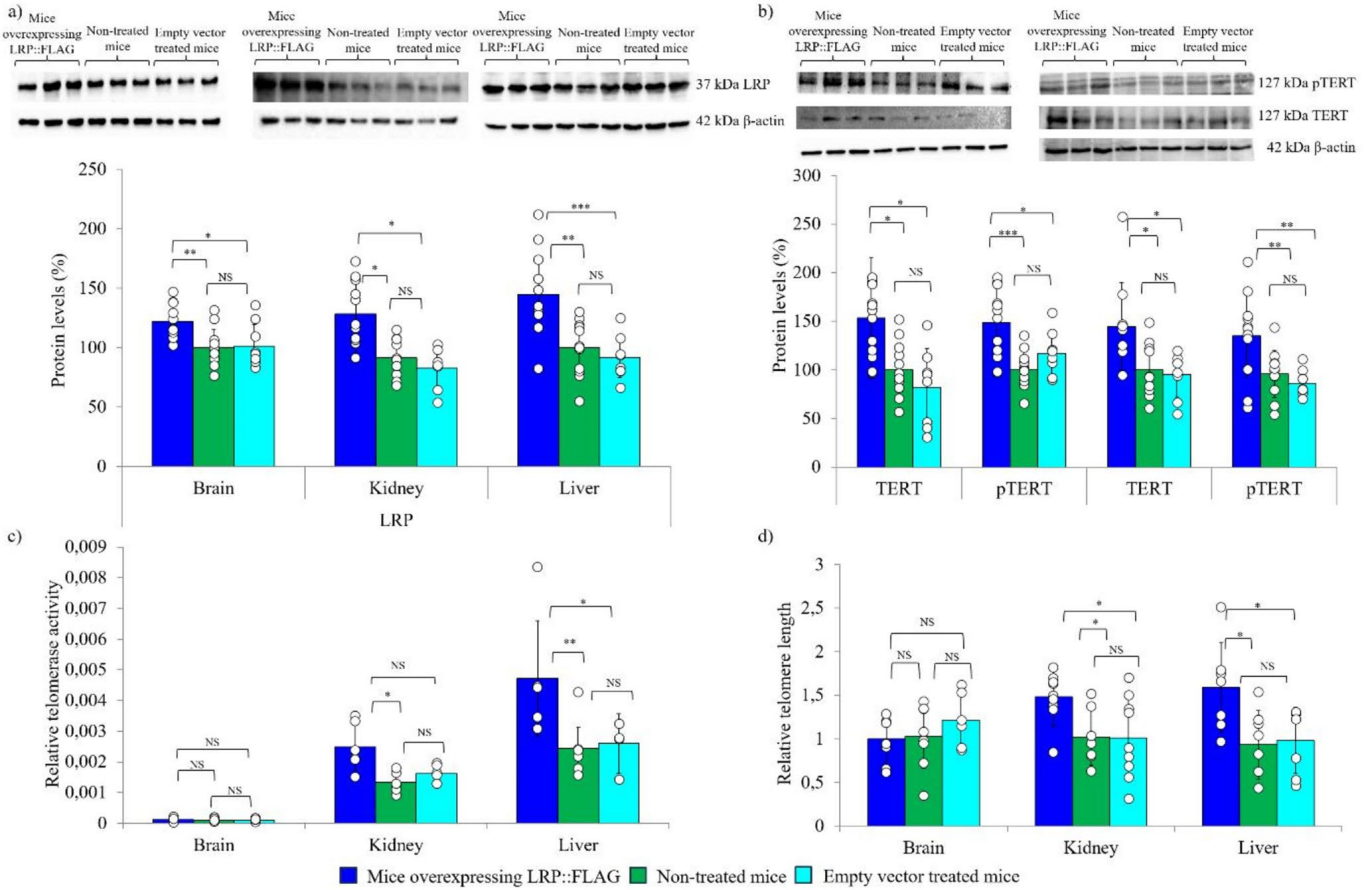
LRP::FLAG overexpression induces a significant increase in TERT levels, telomerase activity and telomere length within aged mice. **a**) LRP::FLAG overexpression was confirmed by immunoblotting and densitometric analysis of total LRP/LR protein levels in the brain, kidney and liver. **b**) Total TERT and phosphorylated TERT (pTERT) protein levels assessed by immunoblotting and densitometry in the brain and liver. Sample size: Brain: Treated: *n* = 11; Control: *n* = 8 and Non-treated: *n* = 12. Liver: Treated: *n* = 10; Control: *n* = 8 and Non-treated: *n* = 10. β-actin was used as a reference protein. All illustrated blots are representative where 4 blots were performed to assess all biological replicates. In addition, a random technical repeat for each treatment was performed on each blot. **c**) Relative telomerase activity was quantified by TRAP and real time qPCR. Telomerase activity values were calculated from the TSR8 standard curve. Data shown for all three organs is representative of sample data subtracted from the heat-treated control. Sample size: Brain: *n* = 7 biological replicates for all three groups. Liver: *n* = 6 biological replicates for treated and non-treated groups while *n* = 5 biological replicates for the control group. Kidney: *n* = 6 biological replicates for all three mouse groups. All samples were run as 2 technical repeats per biological replicate. **d**) Mean telomere length was measured by qPCR and normalised to the reference gene 36B4 to generate a telomere/36B4 (T/S) ratio. Data shown for all three organs is representative of three averaged technical repeats for each biological replicate. Brain: *n* = 7 biological replicates for all three groups. Liver: *n* = 7 biological replicates for treated and non-treated groups while *n* = 6 biological replicates for the control group. Kidney: *n* = 9 biological replicates for treated and non-treated groups while *n* = 7 biological replicates for the control group. Data represented as the mean ± SD. *p < 0.05, **p < 0.01, ***p < 0.001; One-way ANOVA and Welch’s t-test coupled with Bonferroni correction.

The significant improvements noted on a physiological and histological level were further corroborated on a biochemical level. Firstly, LRP::FLAG overexpression was confirmed, where total LRP mRNA and protein levels were quantified. Gene expression analysis for *lrp* (Fig. S2a, Table S2a-c) confirmed that treated mice displayed a significant elevation in gene expression, compared to non-treated and control mice for the kidney (T: 1.85 ± 0.59, C: 1.00 ± 0.36, NT: 1.13 ± 0.59). Similarly, a significant difference was observed between treated and control mice while a trend for improvement following Bonferroni correction was noted between treated and non-treated mice in the liver (T: 1.44 ± 0.557, C: 0.74 ± 0.114, NT: 0.93 ± 0.328), while no significant difference for the brain (T: 1.27 ± 0.23, C: 1.30 ± 0.34, NT: 1.10). Similarly, total LRP protein quantification revealed a significant increase in treated mice for all three tested organs: brain (T: 121.92% ±14.52%, C: 101.12% ±18.16%, NT: 100% ±15.45%), kidney (T: 128.24% ±25.97%, C: 82.62% ±16.30%, NT: 91.51% ±16.31%) and liver (T: 144.91% ±33.06%, C: 91.35% ±18.42%, NT: 99.99% ±22.67%), (Fig. 4a, Table S3a-c), therefore confirming that the pCIneo-moLRP::FLAG vector elevated total LRP/LR levels. The surprising increase in LRP protein levels within the brain tissue could be attributed to the cationic liposomes which have been shown to theoretically be able to traverse the blood brain barrier by absorptive-mediated endocytosis within in vitro models [51]. Alternatively, LRP is known to have a shed isoform (reviewed in [2]), which could have accumulated in the circulatory system and then traversed the blood brain barrier by receptor mediated transport possibly accounting for the elevated LRP in the brain. The discrepancy noted between LRP mRNA expression and protein levels for the brain could be due to a difference in protein turnover rate, differences in mRNA persistence or differences in transcriptional regulation in comparison to other organs such as the kidney or liver.

Upon confirming an increase in total LRP levels, it was prudent to discern whether an effect on TERT, telomerase and telomere length was elicited, as previously noted in vitro [13]. Gene expression analysis by qPCR revealed that treated mice displayed a trend for improvement following Bonferroni correction in mouse *TERT* (*mTERT*) expression in all organs with a significant increase noted in the kidney between treated and non-treated mice [brain (T: 1.4 ± 0.224, C: 1.12 ± 0.502, NT: 1.01 ± 0.566); kidney (T: 2.16 ± 0.95, C: 1.23 ± 0.573, NT: 1.14 ± 0.572); liver (T: 1.64 ± 0.89, C: 1.02 ± 0.352, NT: 1.08 ± 0.433)], (Fig. S2b, Table S2a-c). Similarly, LRP::FLAG overexpression within treated mice induced a significant increase in mTERT protein levels in both the brain (T: 153.36% ±62.44%, C: 82.14% ±39.93%, NT: 100% ±27.77%) and liver (T: 144.70% ±45.55%, C: 94.87% ±22.91%, NT: 100% ±28.11%) compared to control and non-treated mice (Fig. 4b, Table S3a-c). Moreover, treated mice also displayed a greater degree of phosphorylated TERT (pTERT) in both the brain (T: 148.53% ±35.38%, C: 117.20% ±22.80, NT: 100% ±18.35%) and liver (T: 135.15% ±42.60%, C: 86.33% ±12.96%, NT: 95.96% ±24.50%), (Fig. 4b, Table S3a-c). Importantly it must be noted that that there are distinct differences in telomerase and telomere biology between mice and humans. In fact, the majority of mouse tissues exhibit lifelong telomerase activity whilst most human tissues only express basal levels of telomerase activity after early embryonic development [52]. Therefore, the increased TERT expression and protein levels observed following LRP::FLAG overexpression likely carries out extra-telomeric functions such as protecting against oxidative stress and preserving mitochondrial function amongst others, rather than exclusively elevating telomerase activity [53, 54].

In order to confirm whether the increased TERT and pTERT observed caused a subsequent increase in telomerase activity post LRP::FLAG overexpression, a qPCR-based TRAP assay was performed. A significant increase in telomerase activity was observed in the kidney (T: 0.0025 amole ± 0.00075 amole, C: 0.0016 amole ± 0.00033 amole, NT: 0.0013 amole ± 0.00028 amole). Furthermore, a significant difference between treated and untreated mice as well as a trend for improvement post Bonferroni correction was noted between treated and control mice in the liver (T: 0.0047 amole ± 0.0019 amole, C: 0.0026 amole ± 0.00068 amole, NT: 0.0024 amole ± 0.00096 amole) while no significant difference was observed in the brain (T: 0.00012 amole ± 5.67E-05 amole, C: 0.00011 amole ± 4.83E-05 amole, NT: 0.0001 amole ± 4.97E-05 amole), (Fig. 4c, Table S2a-c). Telomere length was assessed by qPCR where the average telomere length of the treated mice was significantly greater when compared to both control and non-treated mice within the liver (T: 1.59 ± 0.514, C: 0.93 ± 0.392, NT: 0.98 ± 0.376) and kidney (T: 1.56 ± 0.167, C: 1.02 ± 0.319, NT: 1.01 ± 0.470) samples, while no significant difference was observed for the brain (T: 1.007 ± 0.26, C: 1.216 ± 0.312, NT: 1.028 ± 0.364), (Fig. 4d, Table S2a-c). This detected increase in telomere length while surprising, considering in-bred mice have exceedingly long telomeres, does coincide with data obtained by de Jesus et al., (2012) who found a change in telomere length in mice from a > 95% C57BL6 background after treatment with AAV9-mTERT vectors utilising Q-FISH [28].

### LRP::FLAG overexpression positively affects proliferative markers and inhibits senescence pathways

**Fig. 5:**
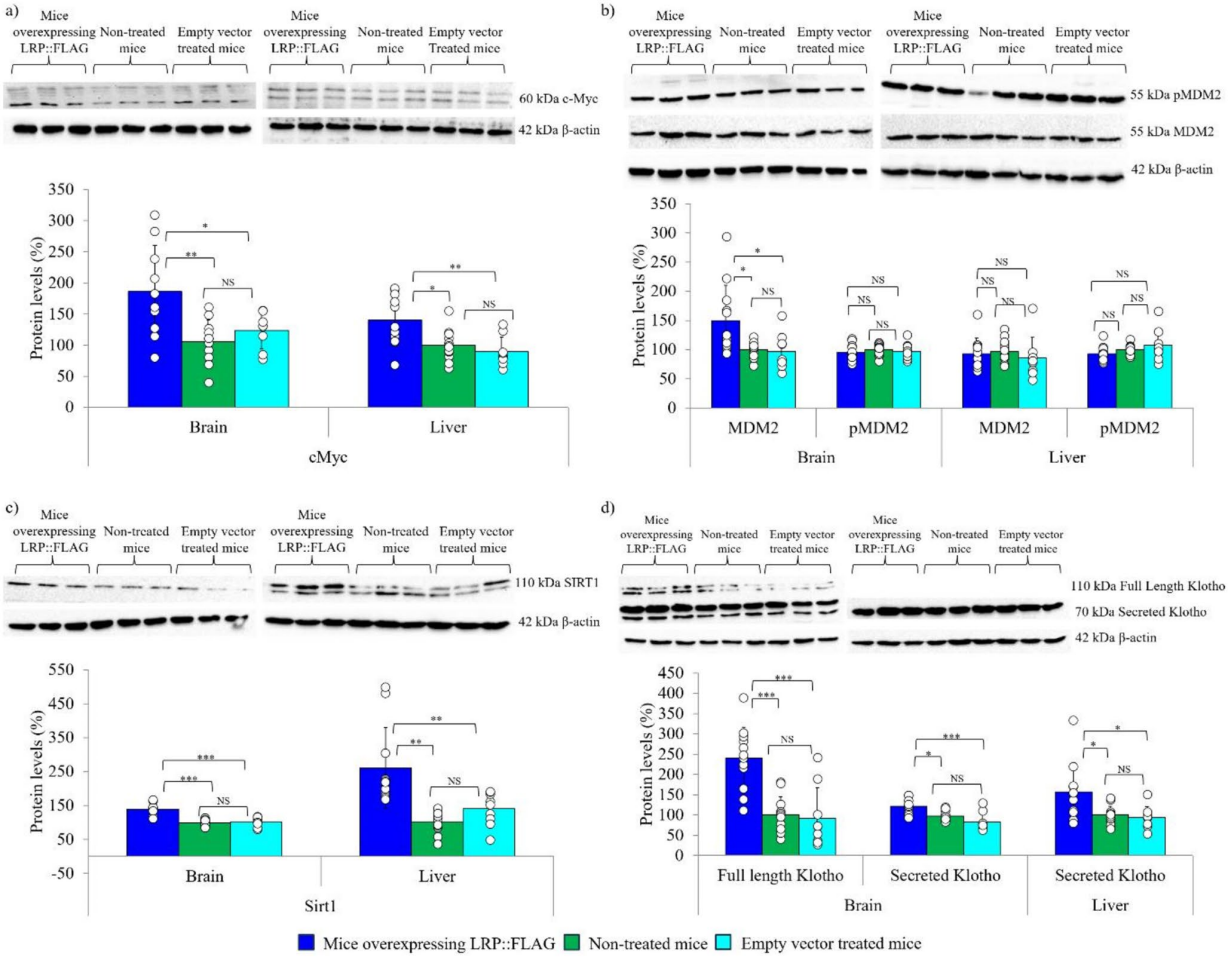
LRP::FLAG overexpression positively affects proliferative and anti-senescence protein markers in aged mice. Following LRP::FLAG overexpression various proliferative and anti-ageing markers were assessed by immunoblot and densitometric analysis. **a**) c-Myc protein levels in the brain and liver. Densitometric analysis was performed on the top band for the liver samples. **b**) MDM2 and pMDM2 protein levels in the brain and liver. **c**) SIRT1 protein levels in the brain and liver. Densitometry was performed on the top band for the liver samples. **d**) Protein levels of both the full length and secreted isoforms of α-klotho were assessed in the brain and liver. In regard to the brain, densitometric analysis was performed on the first set of bands for the full-length isoform, while the third band was quantified for the secreted isoform of α-klotho. As the liver does not produce α-klotho, but should contain secreted/circulating α-klotho the protein levels of secreted klotho were assessed. β-actin was used as a reference protein for all immunoblots. All illustrated blots are representative where 4 blots were performed to assess all biological replicates. In addition, a random technical repeat for each treatment was performed on each blot. Sample size: Brain: Treated: *n* = 11; Control: *n* = 9 and Non-treated: *n* = 12. Liver: Treated: *n* = 11; Control: *n* = 8 and Non-treated: *n* = 12. Kidney: Treated: *n* = 12; Control: *n* = 8 and Non-treated: *n* = 10. (C, D). Data represented as the mean ± SD. *p < 0.05, **p < 0.01, ***p < 0.001; One-way ANOVA and Welch’s t-test coupled with Bonferroni correction

Upon confirming the alterations to telomerase activity and telomere length post LRP::FLAG transfection, it was prudent to assess the downstream effects it exerted on proliferative pathways. Firstly, we assessed the transcription factor (TF) c-Myc, which is involved in TERT and proliferative gene regulation. Gene expression analysis revealed no significant change in *c-myc* expression between treated, control and untreated mouse groups within the brain (T: 1.04 ± 0.3, C: 1.02 ± 0.22, NT: 1.06 ± 0.38). Additionally, a significant difference was noted in the kidney (T: 1.73 ± 0.43, C: 1.24 ± 0.35, NT: 1.01 ± 0.18), while a significant difference between treated and control mice and a trend for improvement following Bonferroni correction was noted between treated and non-treated mice within the liver (T: 1.41 ± 0.372, C: 1.18 ± 0.505, NT: 0.83 ± 0.186), (Fig. S2c, Table S2a-c). Further protein analysis revealed that mice overexpressing LRP::FLAG displayed significantly elevated levels of c-Myc in the brain (T: 185.98% ±74.01%, C: 123.49% ±29.37%, NT: 105.88% ±34.76%) and liver (T: 140.15% ±41.12%, C: 89.51% ±23.64%, NT: 99.99% ±24.67%), (Fig. 5a, Table S3a-c). Next, MDM2 gene expression and protein levels were assessed. MDM2 is an E3 ubiquitin ligase that plays a major role in p53 degradation, thereby regulating p53 functions including: DNA repair, antioxidant and metabolic functions, cell cycle arrest, apoptosis as well as senescence to promote cell cycling and survival [55–59]. Gene expression analysis illustrated no significant change in *mdm2* gene expression between the different mouse groups for the brain (T: 1.12 ± 0.27, C: 1.37 ± 0.11, NT: 1.16 ± 0.38), kidney: (T: 1.29 ± 0.52, C: 1.00 ± 0.45, NT: 0.93 ± 0.35) and liver (T: 1.06 ± 0.33, C: 0.78 ± 0.16, NT: 1.1 ± 0.48), (Fig. S2d, Table S2a-c). Thereafter, total protein level analysis revealed that mice treated to overexpress LRP::FLAG had significantly elevated MDM2 levels within the brain tissue (T: 149.73% ±60.26%, C: 96.65% ±30.72%, NT: 99.99% ±12.98%) whereas no significant difference in MDM2 levels was detected within the liver (T: 92.55% ±27.11%, C: 85.64% ±35.24%, NT: 97.13% ±19.11%), (Fig. 5b, Table S3a-c). Moreover, MDM2 requires phosphorylation to be fully active. Analysis of pMDM2 (pSer166) revealed no significant difference amongst the different treatment groups for both the brain (T: 95.61% ±13.58%, C: 96.5% ±14.22%, NT: 100% ±9.58%) and liver (T: 92.99% ±12.82%, C: 107.24% ±27.09%, NT: 100% ±8.23%) (Fig. 5b, Table S3a-c), confirming that functional cell cycle controls were still in place.

Following the improved proliferative markers, we assessed the effect that LRP::FLAG had on proteins associated with longevity. To this end, SIRT1, a NAD-dependent deacetylase, which plays a regulatory function in DNA repair, inflammation, and the stimulation of TERT and p53 [60, 61], was analysed. Subsequent *sirt1* gene expression analysis revealed a significant elevation in expression within the kidney (T: 1.76 ± 0.794, C: 1.02 ± 0.225, NT: 1.01 ± 0.369) and liver (T: 1.35 0.139, C: 0.92 ± 0.186, NT: 1.03 ± 0.288) of treated mice, while no significant difference was observed for the brain (T: 1.21 ± 0.21, C: 1.162 ± 0.13, NT: 1.11 ± 0.55), (Fig. S2e, Table S2a-c). Furthermore, treated mice demonstrated significantly enhanced SIRT1 protein levels within the brain (T: 139.64% ±15.95%, C: 100.47% ±12.96, NT: 100% ±9.96%) and liver (T: 260.22% ±119.87%, C: 140.35% ±48.48%, NT: 100.42% ±31.59%), (Fig. 5c, Table S3a-c). The next longevity marker assessed was α-klotho, a transmembrane protein that produces a secreted isoform, which influences ageing through insulin signalling, protection against oxidative stress and shares a relationship with longevity proteins, SIRT1 and TERT [62–64]. Accordingly, treated mice (1.86 ± 0.555) exhibited significantly higher *α-klotho* mRNA levels within the kidney compared to non-treated (1.11 ± 0.518) and a trend for improvement post Bonferroni correction compared to control mice (1.24 ± 0.470) (Fig. S2f, Table S2a-c). Furthermore, treated mice (1.33 ± 0.415) exhibited a trend for improvement in *α-klotho* expression compared to non-treated (1.07 ± 0.438) and control mice (1.15 ± 0.239) within the brain tissue (Fig. S2f, Table S2a-c). Protein level analysis verified a significant increase in full length α-klotho within the brain (T: 239.98% ±76.65%, C: 92.23% ±74.95%, NT: 99.99% ±44.73%), as well as in the secreted isoform in both the brain (T: 121.23% ±18.31%, C: 83.50% ±21.78%, NT: 97.63% ±12.69%) and liver (T: 157.22% ±71.38%, C: 94.04% ±26.49%, NT: 100% ±21.85%) of treated mice (Fig. 4d, Table S3a-c).

**Fig. 6:**
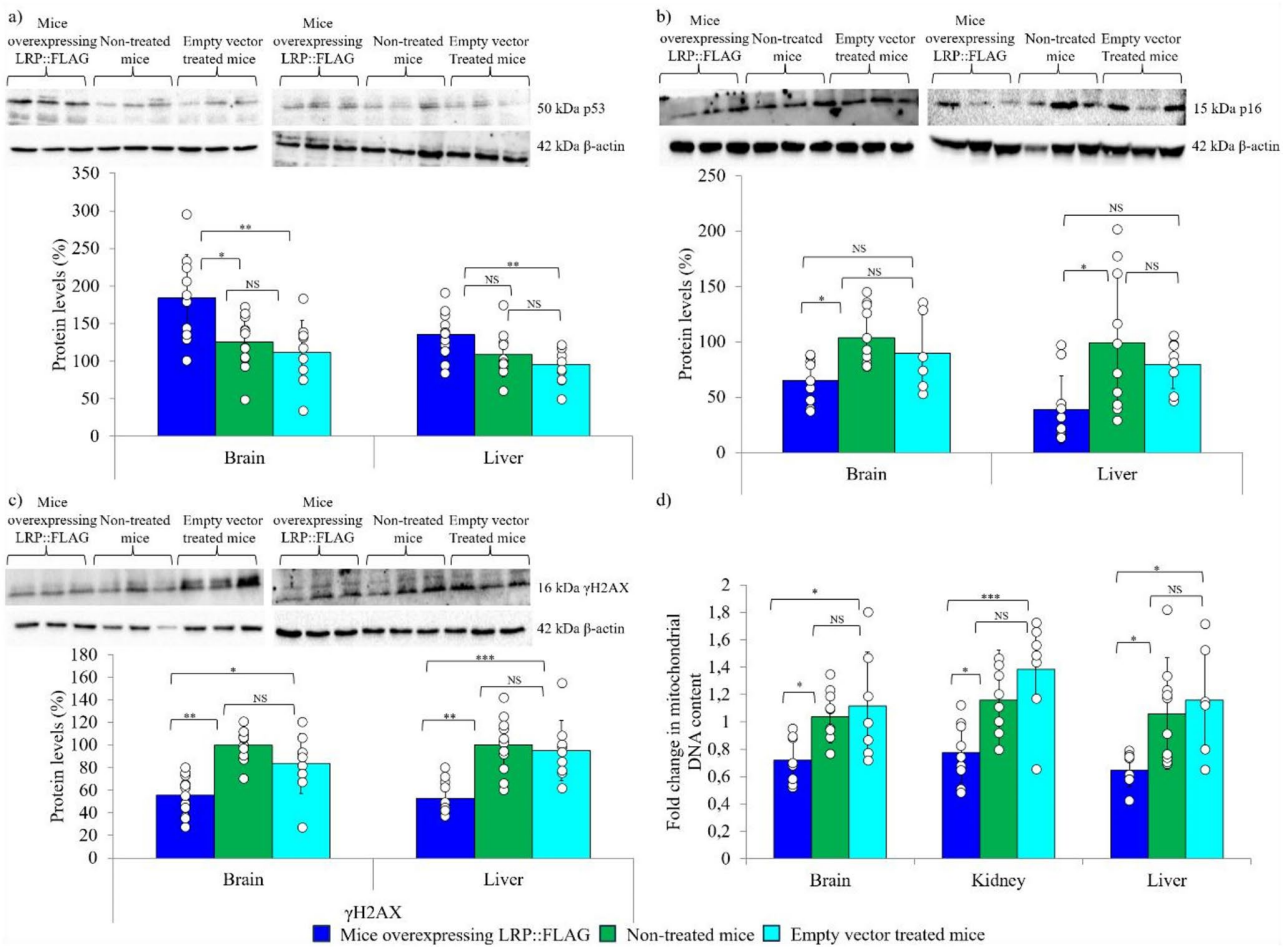
LRP::FLAG overexpression significantly delays the accumulation of senescence markers in aged mice. Immunoblot and subsequent densitometric analysis of **a**) p53, **b**) p16 and **c**) γH2AX protein levels in the brain and liver following LRP::FLAG overexpression. In particular for γH2AX densitometric analysis was performed on the bottom bands. For all immunoblots performed β-actin was used as a reference protein. All illustrated blots are representative where 4 blots were performed to assess all biological replicates. Sample size: Brain: Treated: *n* = 11; Control: *n* = 9 and Non-treated: *n* = 12. Liver: Treated: *n* = 11; Control: *n* = 8 and Non-treated: *n* = 12. Kidney: Treated: *n* = 12; Control: *n* = 8 and Non-treated: *n* = 10. d) Mitochondrial DNA content assessment by qPCR for brain, liver and kidney samples following LRP::FLAG overexpression. Sample size: Brain: Treated: *n* = 8; Control: *n* = 7 and Non-treated: *n* = 11. Liver: Treated: *n* = 8; Control: *n* = 6 and Non-treated: *n* = 10 and Kidney: Treated: *n* = 9; Control: *n* = 7 and Non-treated: *n* = 10. Nuclear specific primers for hexokinase 2 gene, intron 9 were used as reference genes for sample normalisation. Data shown for all three organs is representative of three averaged technical repeats for each biological replicate. Data represented as the mean ± SD. *p < 0.05, **p < 0.01, ***p < 0.001; One-way ANOVA and Welch’s t-test coupled with Bonferroni correction

The effects observed on proliferative and longevity markers warranted the assessment of the effect of LRP::FLAG overexpression on the accumulation of senescence markers. Firstly, p53 was assessed due to its major role in telomere-dependent senescence and since a reduction of this protein is commonly associated with carcinogenesis [reviewed in [58, 65]. Surprisingly, a significant elevation in *p53* levels was noted between treated and control mice while a trend for improvement following Bonferroni correction was noted between treated and non-treated mice within the liver (T: 1.38 ± 0.472, C: 0.75 ± 0.184, NT: 0.94 ± 0.475) and kidney (T: 1.87 ± 0.609, C: 1.29 ± 0.312, NT: 1.18 ± 0.564), while no significant difference was observed in the brain (T: 1.11 ± 0.20, C: 1.19 ± 0.29, NT: 1.18 ± 0.67), (Fig. S3a, Table S2a-c). Furthermore, a significant increase in total p53 protein levels in the brain (T: 183.70% ±58.70%, C: 111.89% ±42.88%, NT: 125.10% ±34.81%) and a significant difference between treated and control mice was observed, while a trend for improvement following Bonferroni correction was noted between treated and non-treated mice within the liver (T: 135.71% ±30.03%, C: 95.80% ±23.09%, NT: 108.55% ±29.71%), (Fig. 6a, Table S3a-c). The next senescence marker assessed was p16INK4A, a protein related to both telomere-dependent and -independent senescence, where its accumulation, especially within mice, has been linked to progression of the ageing phenotype [57, 66]. An overall decrease in *p16* expression within the three tested organs (brain, kidney and liver) was observed (Fig. S3b, Table S2a-c). Thereafter, p16 protein analysis revealed that a significant reduction was evident between treated and non-treated mice, while a decreasing trend following Bonferroni correction was observed between treated and control mice in both the brain (T: 65.11% ±20.52%, C: 89.71% ±35.04%, NT: 103.79% ±26.23%) and liver (T: 38.71% ±30.70%, C: 79.81% ±21.85%, NT: 99.47% ±62.55%), (Fig. 6b, Table S3a-c). An additional factor that influences ageing, namely the accumulation of DNA damage, was assessed utilising the DNA damage marker γH2AX, which accumulates with age [67]. Protein analysis indicated a significant reduction in γH2AX levels in the brain (T: 55.73% ±17.24%, C: 88.88% ±35.04%, NT: 100% ±13.86%) and liver (T: 52.86% ±14.02%, C: 95.20% ±26.69%, NT: 100.18% ±23.71%) of the treated mice (Fig. 6c, Table S3a-c). Finally, mitochondrial dysfunction was assessed by qPCR, as most age-related diseases exhibit compromised mitochondria. Treated mice contained significantly less mitochondrial DNA in the brain (T: 0.72 ± 0.17, C: 1.12 ± 0.398, NT: 1.04 ± 0.187), kidney (T: 0.78 ± 0.22, C: 1.39 ± 0.369, NT: 1.06 ± 0.301) and liver (T: 0.65 ± 0.12, C: 1.16 ± 0.408, NT: 1.06 ± 0.363), compared to non-treated and control mice (Fig. 5d, Table S2a-c). Together, these data illustrate that LRP::FLAG overexpression may delay senescence onset.

## Discussion

The research pertaining to healthy ageing and longevity have been a prime target of scientific investigation for decades, due to the enticing prospect of increasing the average human lifespan. However, an additional factor that has stimulated increased research motivation into this field, is the ever-increasing number of diseases shown to be tied to ageing. The current study serves to demonstrate that LRP/LR could stimulate TERT/telomerase, and may harbour other anti-ageing properties, due to its apparent broad spanning effect on other senescence and anti-ageing related proteins. The marked decrease in hair greying, thinning and balding of treated mice (Fig. 1a1,2) suggests that the hair follicles in treated mice, were lost at a reduced rate by delayed senescence onset. Additionally, C57BL/6J mouse strain have been confirmed to exhibit motor skill deficits with increased age [44, 45, 68], which is potentially brought about by the loss in muscle fibres from muscular atrophy, low grade persistent inflammation with a corresponding reduced energy production due to mitochondrial dysfunction [46, 69]. The stark improvements in motor function and balance (Fig. 1b, c). that treated mice displayed, therefore suggests that LRP::FLAG overexpression may aid in delaying the age-dependent decline of motor function.

Coinciding with these improved physiological aspects, it was noted that LRP::FLAG overexpression in aged mice improved cognitive function. With normal ageing there is a corresponding reduction in cognitive function brought about by a loss in neuronal tissue, especially within the hippocampus, and negative alterations to neuro-based signalling pathways [70]. From the results obtained, it was evident that LRP::FLAG overexpression had a profound effect, as treated mice had superior short term memory (puzzle box test and 2 h NOR), long term memory (puzzle box test and 24 h NOR), puzzle solving ability (puzzle box test) and spatial memory (nesting test) compared to their non-treated and control counterparts (Fig. 2a, c,d). This implied that treated mice showed greater spatial recognition and reference memory, suggesting that neural plasticity within the hippocampus was maintained [48], and age-dependent neurodegeneration of the hippocampus was delayed.

This was corroborated by widespread improvements to tissue structure noted during histochemical analysis (Fig. 3). Brain tissue of the mice overexpressing LRP::FLAG, specifically the hippocampus, was found to be more defined with fewer tissue abnormalities (Fig. 3a1,2). More specifically, treated mice showed less vacuolation and pyknotic nuclei, as well as a larger number of cells spanning the width of the DG compared to control and non-treated mice. This could be indicative that the increased LRP was either preventing apoptosis within these neuronal cells or that neurogenesis was enhanced within the DG to delay the effects of neurological ageing [71]. Collectively, these morphological features within the neuronal tissue of the mice indicate a senescing hippocampus for control and non-treated mice, while treated mice displayed healthier neuronal tissue. Additionally, kidney tissue commonly develops various age-related tissue pathologies within the C57BL/6J mouse strain, including increased glomerulosclerosis [49, 50]. Assessment of non-treated and control mice showed a greater accumulation of pathological markers compared to treated mice (Fig. 3b1,2). Moreover, a subset of control and non-treated mice also displayed severe atrophy and replacement of the glomerulus tissue with fibrotic tissue (Fig. 3b1). This suggests that LRP::FLAG overexpression may slow-down renal tissue senescence and delay the onset of age-dependent decline of the kidneys.

The improved physiological and histological parameters observed post LRP::FLAG overexpression was likely a consequence of the changes observed in proliferative/ survival-related proteins (c-Myc, MDM2), anti-ageing proteins (SIRT1, Klotho), senescence markers (p53, γH2AX, p16), as well as telomerase activity and telomere length (Figs. 4, 6 and 5) [72–74]. The validation of in vitro findings that LRP::FLAG overexpression positively influenced telomere length through telomerase are very important. Although the dynamic that governs telomere biology in ageing is different between humans and mice, treatments that modulate telomerase and the telomeres still produce comparable effects, especially for healthier ageing in humans and mice [75, 76]. LRP::FLAG overexpression not only caused a significant increase in mTERT and pTERT protein levels, but also stimulated its expression at a gene level (Fig. 4b; Fig. S2b). This would suggest that LRP may have the potential to stimulate TERT expression through the activation of appropriate signalling cascades like MAPK, PI3K-Akt or Wnt to delay the senescence process [77, 78]. This elevation in TERT, besides allowing it to perform additional telomeric extension, may also allow for enhanced extra-telomeric functioning.

Further supporting this theory, was the elevated gene and protein expression levels of the transcription factor c-Myc (Fig. 5a; Fig. S2c), which not only indicated an increase in Wnt proliferative and survival signalling, but further validated the increase in TERT expression [79–83]. Alongside the increased levels of TERT and its phosphorylated isoform, there was a corresponding elevation in telomerase activity and mean telomere length within the kidney and liver following LRP::FLAG overexpression (Fig. 4c, d). Therefore, corroborating the in vitro findings that altering LRP levels can be utilised to modulate telomerase activity and telomere length to delay the senescence process [14]. Adult mice display basal telomerase activity within the brain since the majority of the TERT expressed is involved in extra-telomeric functions, which explains the lack of change in telomerase activity and telomere length noted [84, 85]. Additionally, the decreased mtDNA content (Fig. 6d) indicates that the elevated TERT may have aided in preserving mitochondrial function and protected the mitochondria against oxidative damage [79].

Mitochondrial dysfunction is common during ageing and age-related diseases. This is characterised by deterioration in mitochondrial respiratory function, which heightens ROS production and damages mtDNA, thereby causing mutations and mitochondrial mass to accumulate [86–90]. This increases oxidative stress causing extensive DNA, lipid and protein damage, which accelerates senescence [91]. In the mitochondria, TERT plays a dual extra-telomeric role, where it protects mtDNA from oxidative damage [53, 54, 79, 92, 93]. However, if the damage is too extensive, TERT can help initiate mitophagy for its removal [79, 92, 93]. Masuyama et al. (2005) [94] showed an increase in brain and kidney mtDNA content as mice age, and Correia et al. (2016) [95], found that reductions in mtDNA copy number prevented tissue senescence within the liver in vivo. Our data corroborates these observations, and we suggest that the reduced mtDNA content observed in mice overexpressing LRP::FLAG was due to selective degradation of damaged mitochondria, to reduce mitochondrial dysfunction (Fig. 6d). Furthermore, the reductions in IL6 and IL8 (Fig. S3c-d, Table S2a-c) in the brain and kidney of treated mice, potentially indicate reduced inflammation and oxidative stress. Together, these data illustrate that LRP::FLAG overexpression may delay senescence onset.

Advanced ageing is correlated with an overall lack in proliferation caused by an imbalance between growth promoting and growth inhibitory signalling, culminating in the loss of tissue replenishment, whereas restoring controlled and active proliferative signalling has been associated with tissue restoration [72–74]. In ageing, the p53-MDM2 axis is often modified to favour p53 elevation to induce either early or delayed ageing onset, depending on the presence of other senescence markers [96]. Our study revealed that LRP::FLAG overexpression induced no difference in *mdm2* gene expression (Fig. S2d) or MDM2 protein levels within the liver samples (Fig. 5b). This could be attributed to the liver being a highly regenerative organ, which displays higher basal levels of MDM2. In contrast, the brain displayed elevated levels of MDM2 within treated mice (Fig. 5b), which could be a consequence of the increased p53 observed, to maintain the p53-MDM2 axis for proper functioning [96]. In accordance, no significant increase in pMDM2 (pSer166), which signals MDM2 to bind p53 for ubiquitination and proteasomal degradation, (Fig. 5b) was noted for either organ [85]. Therefore, suggesting that proper cell cycle controls were in place to prevent exhaustive proliferation and tumorigenic development [96, 97].

A surprising finding was the elevation in p53 protein levels and gene expression following LRP::FLAG overexpression (Fig. 6a, Fig. S3a). This tumour suppressor has broad spanning roles in cancer, apoptosis, DNA repair, and senescence (reviewed in [58]). Although a marked elevation in p53 is normally associated with telomere-dependent senescence; research has shown that p53 induces elimination of cells exhibiting telomere damage, rather than promoting senescence [48]. Therefore, we propose that the increased p53 observed promotes longevity. Prior published findings showed that increased p53, alongside p19ARF in mice, heightens resistance to tumour development, with a significant increase in longevity [55]. One explanation for the increased p53 in the treated mice is that the elevated LRP levels may have stimulated p53 expression to act as a compensatory mechanism to ensure LRPs proliferative and survival functionality was tightly regulated. Alternatively, it has been shown that ribosomal proteins are capable of binding to MDM2 to allow for p53 stabilization. Due to the fact that LRP also functions as a ribosomal protein it may have the ability to bind MDM2 for p53 regulation [59] This would have ensured that the proliferative and potential anti-ageing functions of LRP were tightly regulated to protect against potential tumorigenic onset.

In addition to LRP/LR’s suggested anti-senescence functionality, it was able to positively influence proteins directly involved in anti-ageing and longevity responses. The increase in both gene expression and protein levels of SIRT1 following LRP::FLAG overexpression (Fig. 5c, Fig. S2e), indicates a delay of the senescence process. SIRT1 plays a regulatory role in gene expression and in subsequent signalling cascades for several proteins, including TERT, which influences DNA repair, metabolism and longevity processes [60, 61]. Additionally, SIRT1 is implicated in reducing oxidative stress and inflammation, and acts as a tumour suppressor [98–100]. Aside from reductions in γH2AX, indicating reduced DNA damage in the treated mice, it is further suggested that the elevated SIRT1 aided in maintaining telomere length either by influencing telomerase activity or affecting the telomeres [101]. In addition, the increased SIRT1 may have aided in reducing the senescence-related inflammatory markers IL6 and 8 (Fig. S3c, d) in kidney and brain, by deacetylating and inactivating NFκB [102, 103]. The interconnections and positive effects presented by the data, indicate that elevated LRP increases the anti-ageing protein SIRT1 through post-translational processes or by influencing signalling cascades to bring about an anti-senescence effect for healthier ageing.

The second anti-ageing protein that LRP::FLAG was found to influence was α-klotho, a transmembrane protein that is found in three isoforms (full length, shed and secreted) [64]. This protein is predominantly produced in the kidney and brain [104] and its dysregulation can induce widespread and systemic effects to aggravate the natural ageing processes or the development of age-related diseases [105, 106]. In fact, dysregulation of this protein has been associated with accelerated ageing phenotypes due to elevations in ROS production, insulin signalling and mitochondrial dysfunction [95–97]. Mice overexpressing LRP::FLAG exhibited increased α-klotho gene expression (Fig. S2f) in the kidney, with an increase in both full length and secreted klotho protein in the brain, and an increase in secreted α-klotho in the liver (Fig. 5d). This coincides with the increased SIRT1, to suggest an anti-senescence effect occurred [63].

These findings are further substantiated by the stark decrease in p16 protein levels (Fig. 6b). Since mice exhibit a lack of critically shortened telomeres throughout their lifespan, ageing is caused by a stress-dependent senescence process which is regulated by p16 as the master regulator of senescence [107, 108]. In addition, the reduction of p16 within senescent cells has been found to reverse the ageing phenotype [66, 109]. This study aligns with this, as the decreased gene expression and protein levels of p16 appeared to positively affect tissue and organism fitness. DNA damage accumulation is one of the lead driving forces in ageing. In response to the presence of DSBs, H2AX (H2A variant) is phosphorylated to elicit a DNA damage response [67, 110]. During ageing, senescent cells accumulate γH2AX that eventually form foci, which are resistant to repair [111]. Due to critical telomere erosion or dysfunction, these foci accumulate at telomeric ends and within genomic DNA, and eventually induce a state of permanent cell cycle arrest typical of senescence [111]. The significant reduction in γH2AX alongside the increase in mean telomere length following LRP::FLAG overexpression (Fig. 6c) therefore clearly suggests an anti-senescence effect.

Although the data generated from this study offers novel insights, we have identified several study limitations which could be addressed in future studies. Firstly, the study only focused on one specific strain and sex (female) of mice, where future studies could expand and ensure that the effects observed occur in both sexes and across different strains. Likewise, research could be directed to determine whether these effects occur in other experimental animal models. In addition, a larger sample size could be used to confirm all the effects in a larger cohort and allow for enhanced statistical representation. Another important aspect to consider in future would be to conduct a longevity-based study to determine if there are effects on longevity or if modulation of LRP just induces a healthier form of ageing. Future studies could explore the duration of therapeutic response following the transfection procedure. Lastly, although an increase in LRP was confirmed in several organs indicating a successful transfection, the transfection efficiency in terms of the exact distribution and number of cells within a particular organ that were transfected was not established. This could be evaluated in future by utilising a reporter molecule such as GFP attached to LRP and imaging tissue sections to ensure accurate transfection efficiency determination. In conclusion, these data from both the in vitro [13] and in vivo studies clearly implicate LRP in a novel role within the process of cellular ageing. This role appears to be centred around modulating TERT and telomerase activity levels to influence telomere length and anti-ageing proteins, as well as aiding in preventing the accumulation of senescence markers. This study offers valuable insight into the process of cellular ageing and further presents the potential for alternative therapeutic design. Therefore, strategies aimed at targeting or elevating LRP may well provide an alternative therapeutic strategy for delaying the effects of ageing and potentially treat age-related diseases.

## Electronic supplementary material

Below is the link to the electronic supplementary material.

Supplementary Material

## Data Availability

The data that support the findings of this study are available from the corresponding author [eloise.vandermerwe@wits.ac.za] upon reasonable request.

## Abbreviations

36B4: Acidic ribosomal phosphoprotein
µg: Microgram
µl: Microliter
µm: Micrometre
Aβ: Amyloid beta
BCA: Bicinchoninic acid
BSA: Bovine serum albumin
cDNA: Complimentary deoxyribonucleic acid
CHAPS: 3-((3-cholamidopropyl) dimethylammonio)-1-propanesulfonate
cm: Centimetre
c-Myc: Myelocytomatosis oncogene
DDR: DNA damage response
DG: Dentate gyrus
dH_2_O: Distilled water
DNA: Deoxyribonucleic acid
DNase: Deoxyribonuclease
DSB: Double stranded breaks
EGTA: Ethylene glycol-bis (β-aminoethyl ether)-N, N,N′,N′-tetraacetic acid
g: Gram
gDNA: Genomic DNA
H2AX: H2A histone family member X
HEK293: Human embryonic kidney
H: Hour
hTERT: Human telomerase reverse transcriptase
IL: Interleukin
kDa: Kilodalton
LRP: Laminin receptor precursor
LRP/LR: 37 kDa laminin receptor precursor/67 kDa high affinity laminin receptor
m: metre
MAPK: Mitogen activated protein kinase
MDM2: Mouse double minute 2
mg: Milligram
min: Minute
ml: Millilitre
mM: Millimolar
mm: Millimetre
MRC-5: Medical Research Council cell strain 5
mRNA: Messenger RNA
mtDNA: Mitochondrial Deoxyribonucleic Acid
mTERT: Mouse telomerase reverse transcriptase
NAD: Nicotinamide adenine dinucleotide
NFκB: Nuclear factor kappa-light-chain-enhancer of activated B cells
ng: Nanogram
NOR: Novel object recognition
PBS: Phosphate buffered saline
PBST: Phosphate buffered saline with tween
PCR: Polymerase chain reaction
PFA: Paraformaldehyde
pTERT: Phosphorylated TERT
PVDF: Polyvinylidene fluoride
qPCR: Quantitative polymerase chain reaction
RIPA: Radioimmunoprecipitation assay
RNA: Ribonucleic acid
RNase: Ribonuclease
ROS: Reactive oxygen species
RPSA: Ribosomal protein SA
RT: Room temperature
SDS-PAGE: Sodium dodecyl sulphate– polyacrylamide gel electrophoresis
sec: Seconds
SIRT1: Sirtuin 1
SYBR: N’,N’-dimethyl-N-[4-[(E)-(3-methyl-1,3-benzothiazol-2-ylidene)methyl]-1-phenylquinolin-1-ium-2-yl]-N-propylpropane-1,3-diamine
TERC: Telomerase RNA component
TERT: Telomerase reverse transcriptase
TF: Transcription factor
TRAP: Telomeric repeat amplification protocol
V: Volt
v/v: Volume/volume
W: Watt
w/v: Weight/volume
Wnt: Wingless/Integrated pathway
xg: Times gravity
