# Supplementary material for: 37 kDa LRP::FLAG enhances telomerase activity and reduces ageing markers in vivo
Source: Cell Mol Life Sci. 2025 Feb 22;82(1):83. doi: 10.1007/s00018-025-05593-0 (PMC11846807; doi:10.1007/s00018-025-05593-0)
Supplement: Supplementary file 1 — Supplementary Material [file 18_2025_5593_MOESM2_ESM.docx]

**37 kDa LRP::FLAG enhances telomerase activity and reduces ageing markers *in vivo***


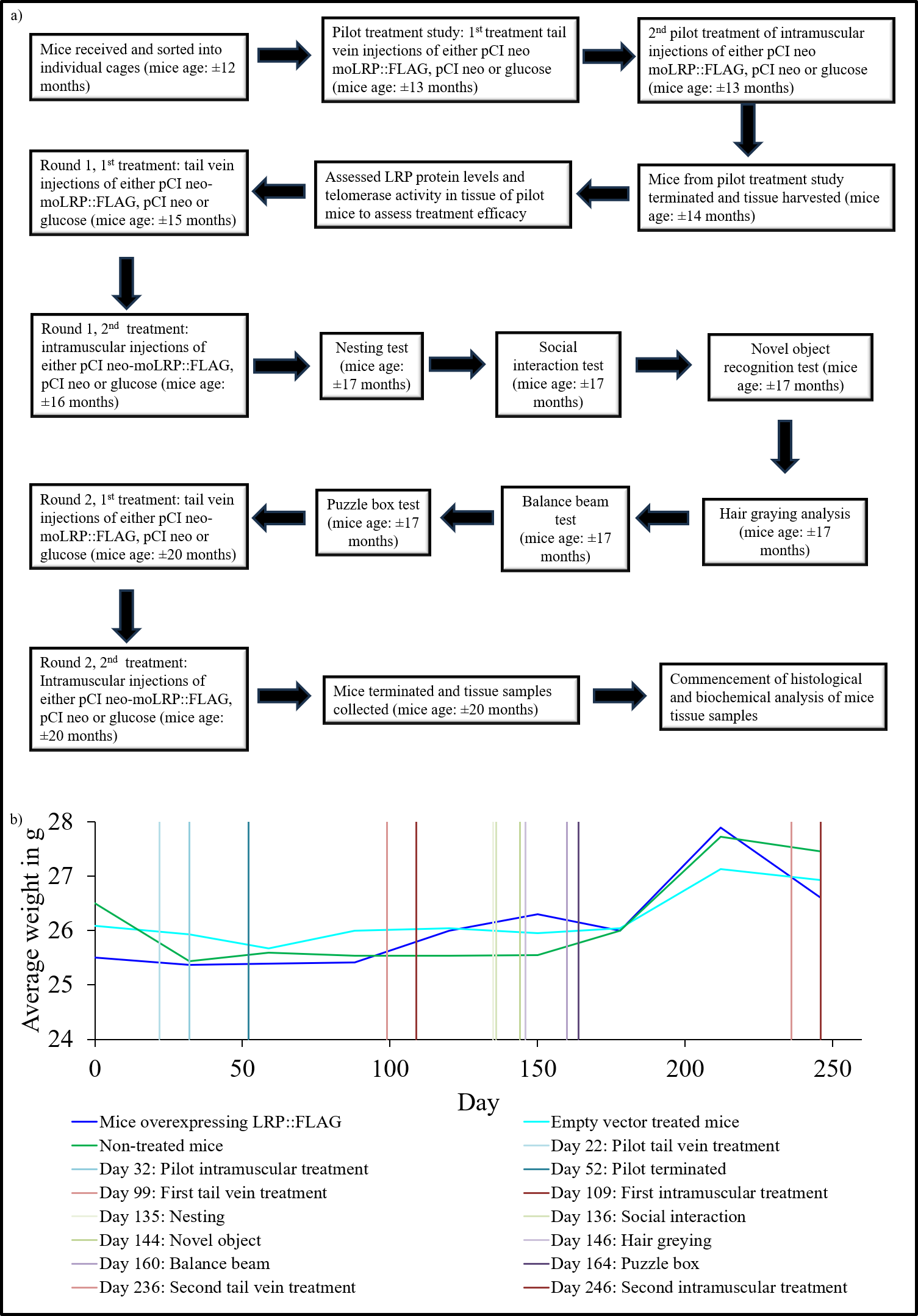
**Supplementary Data**

**Figure S1: Overview of experimental workflow and the corresponding average weight of the mice during each experimental procedure.** a) Schematic flow diagram detailing the different experimental procedures and the order that they were conducted on the mice. The approximate age of the mice at the time of each procedure has also been provided in brackets. b) Analysis of the average weights of the mice from the treated, control and non-treated groups. Lines intersecting graph indicate timepoints for when each different experimental procedure was carried. Sample size at start of study: mice overexpressing LRP::FLAG: n=12; non-treated mice: n=12; empty vector treated mice: n=12.

**
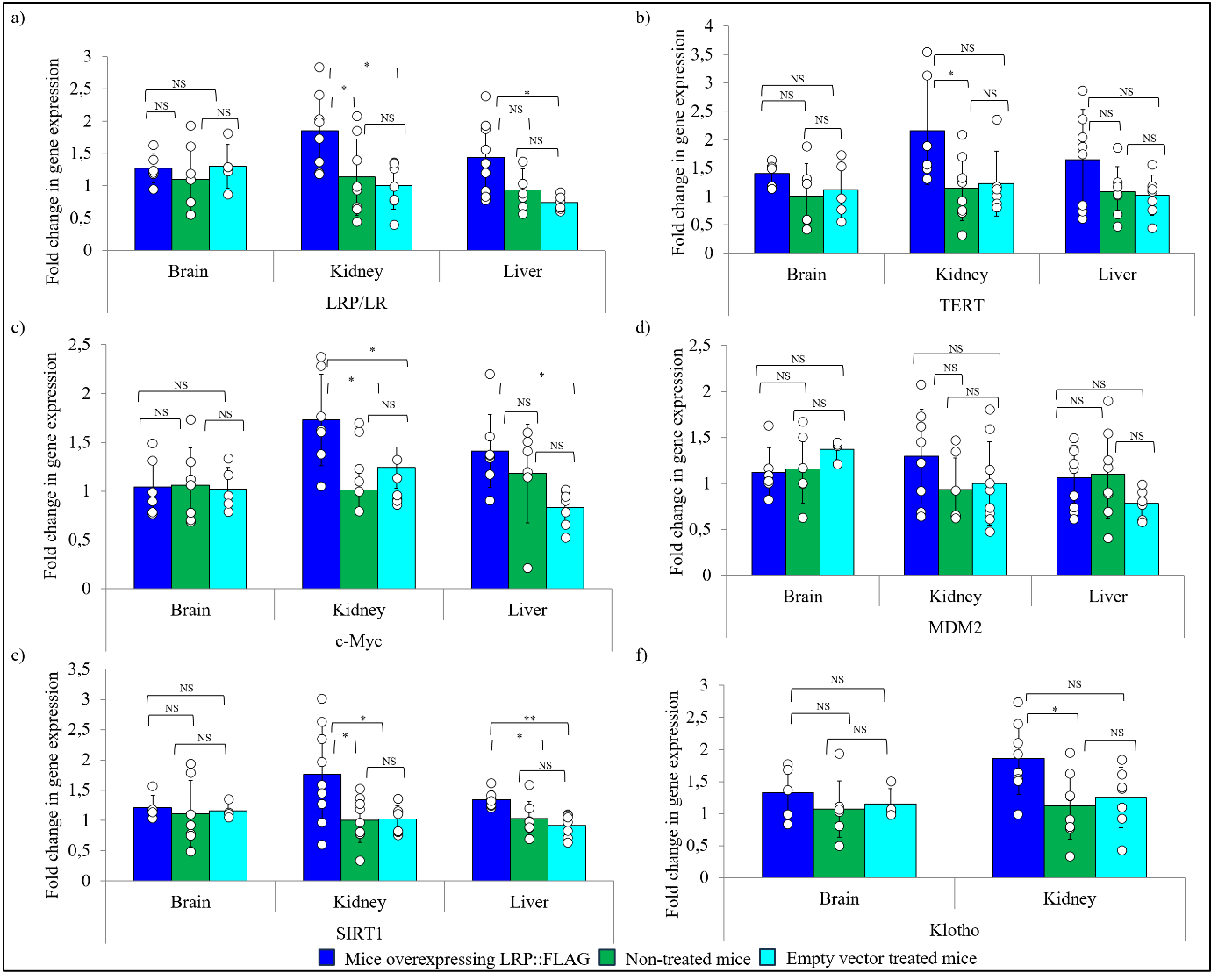
**

**Figure S2: LRP::FLAG overexpression positively affects gene expression of proliferative and anti-ageing markers in aged mice.** Gene expression analysis by qPCR of: a) *LRP/LR,* b) *TERT*, c) *c-Myc*, d) *MDM2*, e) *SIRT1* and f) α-*Klotho* gene expression levels in the brain, liver and kidney were compared between the different mouse treatment groups. a) *LRP/LR* sample size: Treated: n = 5; Control: n = 5 and Non-treated mice: n = 6; Liver: Treated: n = 9; control: n = 6 and Non-treated: n = 7; Kidney: Treated: n = 8; Control: n = 7 and Non-treated: n = 8. b) *TERT* sample size for: Brain: Treated: n = 5; Control: n = 5 and Non-treated: n = 6. Liver: Treated: n = 8; Control: n = 7 and Non-treated: n = 7. Kidney: Treated: n = 6; Control: n = 6 and Non-treated: n = 8. c) *c-Myc* sample size: Brain: Treated: n = 6; Control: n = 5 and Non-treated: n = 7. Liver: Treated: n = 8; Control: n = 7 and Non-treated: n = 6. Kidney: Treated: n = 7; Control: n = 6 and Non-treated: n = 6. d) *MDM2* sample size: Brain: Treated: n = 6; Control: n = 4 and Non-treated: n = 6. Liver: Treated: n = 9; Control: n = 6 and Non-treated: n = 8. Kidney: Treated: n = 8; Control: n = 7 and Non-treated: n = 9. e) ) *SIRT1* sample size: Brain: Treated: n = 6; Control: n = 4 and Non-treated: n = 6. Liver: Treated: n = 7; Control: n = 7 and Non-treated: n = 7. Kidney: Treated: n = 9; Control: n = 7 and Non-treated: n = 8. b) α-*Klotho* sample size: Brain: Treated: n = 5; Control: n = 4 and Non-treated: n = 7. Kidney: Treated: n = 8; Control: n = 7 and Non-treated: n = 8. GAPDH and RPLPO were used as reference genes for sample normalisation. Data represented as the mean ±SD. *p < 0.05, **p < 0.01, ***p < 0.001; One-way ANOVA and Welch’s *t-test* coupled with Bonferroni correction.


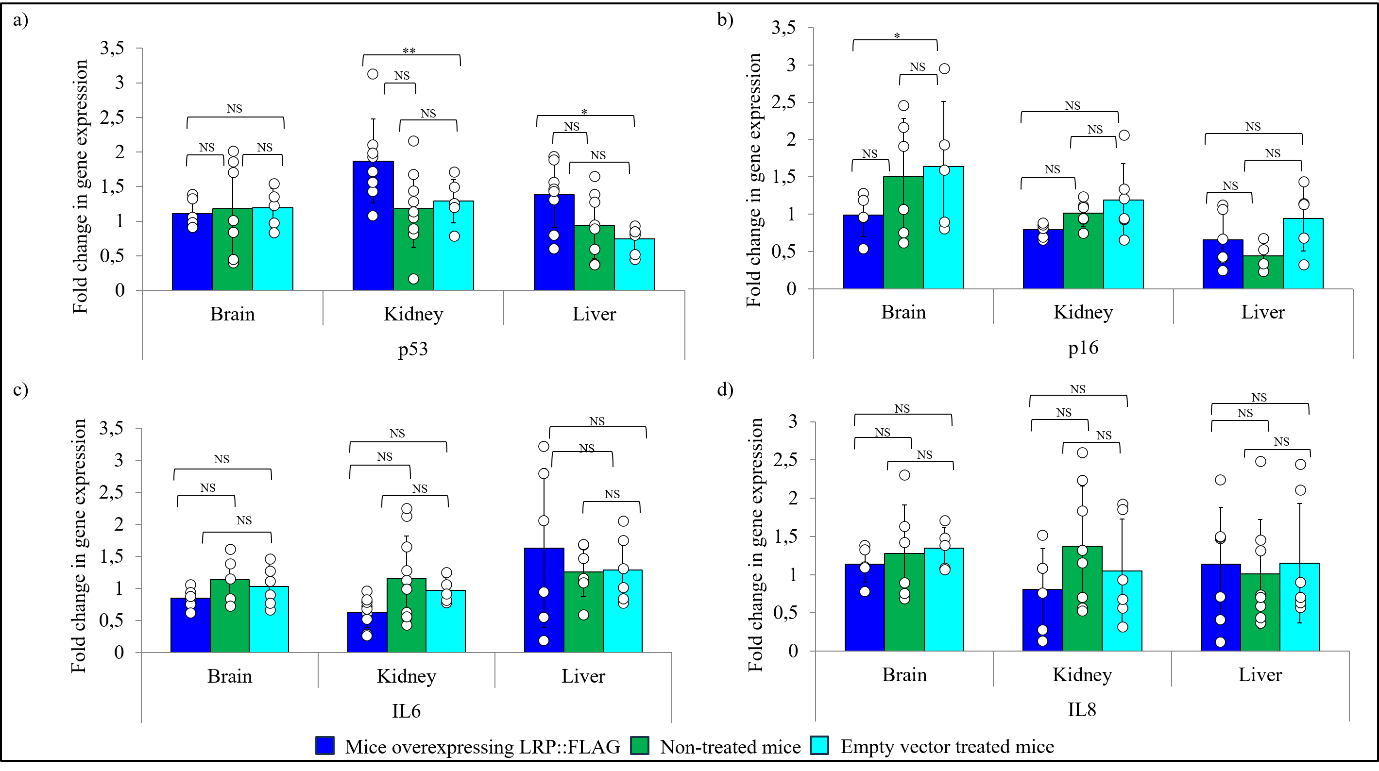


**Figure S3: LRP::FLAG overexpression reduces the gene expression of senescent markers in aged mice.** Gene expression analysis by qPCR of: a) *p53,* b) *p16,* c) *IL6* and d) *IL8* in the brain, liver and kidney were compared between the different mouse treatment groups. a) *p53* sample size: Brain: Treated: n = 6; Control: n = 5 and Non-treated: n = 7. Liver: Treated: n = 8; Control: n = 7 and Non-treated: n = 8. Kidney: Treated: n = 8; Control: n = 6 and Non-treated: n = 9. b) *p16* sample size: Brain: Treated: n = 5; Control: n = 5 and Non-treated: n = 6. Liver: Treated: n = 6; Control: n = 4 and Non-treated: n = 7. Kidney: Treated: n = 6; Control: n = 6 and Non-treated: n = 6. c) *IL6* sample size: Brain: Treated: n = 6; Control: n = 5 and Non-treated: n = 6. Liver: Treated: n = 6; Control: n = 6 and Non-treated: n = 7 and Kidney: Treated: n = 9; Control: n = 5 and Non-treated: n = 10. d) *IL8* sample size: Brain: Treated: n = 5; Control: n = 5 and Non-treated: n = 6. Liver: Treated: n = 7; Control: n = 7 and Non-treated: n = 8 and Kidney: Treated: n = 6; Control: n = 6 and Non-treated: n = 8. GAPDH and RPLPO were used as reference genes for sample normalisation. Data represented as the mean ±SD. *p < 0.05, **p < 0.01, ***p < 0.001; One-way ANOVA and Welch’s *t-test* coupled with Bonferroni correction.

**Tables:**

**Table S1: Statistical analysis for physiological and histological data**

| **Physiological and Histological analysis** | **Anova** | **Mice overexpressing LRP::FLAG** | **Control mice** | **Non-treated mice** |
| --- | --- | --- | --- | --- |
| Hair greying | p = 0.004 | Mean = 25.38%,  SD = 14.11% | Mean = 44.42%,  SD = 10.58% | Mean = 46.68%,  SD = 12.59% |
| Balance beam (Time) | p = 0.0001 | Mean = 16.375 sec,  SD = 4.14 sec | Mean = 26.813 sec  SD = 3.90 sec,  p = 0.0001 | Mean = 25 sec  SD = 4.43 sec,  p = 0.0013 |
| Balance beam (Score) | p = 4.18E-05 | Mean = 4.25,  SD = 0.775 | Mean = 28.125,  SD = 0.403,  p = 2.75E-07 | Mean = 2.625,  SD = 0.72,  p = 9.15E-07 |
| Social interaction | p = 0.432 | Object:  Mean = 33.50%  Caged mouse:  Mean = 66.50%,  SD = 4,81% | Object:  Mean = 35.92%  Caged mouse:  Mean = 64.08%,  SD = 8,51% | Object:  Mean = 38.98%  Caged mouse:  Mean = 61.02%,  SD = 6.91% |
| Novel object recognition 2hr | p = 0.0007 | Old object:  Mean = 36.19%  Novel object:  Mean = 63.81%,  SD = 10.77% | Old object:  Mean = 57.50%  Novel object:  Mean = 42.50%,  SD = 14.46%,  p = 0.0015 | Old object:  Mean = 52.42%  Novel object:  Mean = 47.58%,  SD = 8.77%,  p = 0.0012 |
| Novel object recognition 24hr | p = 0.096 | Old object:  Mean = 40.62%  Novel object:  Mean = 59.38%,  SD = 16.28% | Old object:  Mean = 51.16%  Novel object:  Mean = 48.84%,  SD = 19.5%,  p = 0.188 | Old object:  Mean = 57,97%  Novel object:  Mean = 42.03%,  SD = 17.27%,  p = 0.02 |
| Nesting | p = 0.0003 | Mean = 4.89,  SD = 0.782 | Mean = 3.2,  SD = 1.03,  p = 0.00096 | Mean = 3.2,  SD = 0.79,  p = 0.00022 |
| Brain histology | p = 0.001 | Mean = 14.63,  SD = 2.56 | Mean = 11.5,  SD = 1.38,  p = 0.013484 | Mean = 10.25,  SD = 1.67,  p = 0.001602 |
| Kidney histology | p = 0.001 | Mean = 29.66%,  SD = 6.31% | Mean = 47.08%,  SD = 9.13%,  p = 0.00243 | Mean = 44.20%,  SD = 9.10%,  p = 0.0022 |

**Table S2a: Statistical analysis for gene expression in the mice brain tissue**

| **Brain** | | | | |
| --- | --- | --- | --- | --- |
| **Genes analysed** | **Anova** | **Mice overexpressing LRP::FLAG** | **Control mice** | **Non-treated mice** |
| LRP/LR | p = 0.6454 | Mean = 1.27,  SD = 0.23 | Mean = 1.30,  SD = 0.34, p = 0.88 | Mean = 1.10,  SD = 0.53, p = 0.46 |
| c-MYC | p = 0.9818 | Mean = 1.04,  SD = 0.3 | Mean = 1.02,  SD = 0.22 | Mean = 1.06,  SD = 0.38 |
| MDM2 | p = 0.4097 | Mean = 1.12,  SD = 0.27 | Mean = 1.37,  SD = 0.11 | Mean = 1.16,  SD = 0.38 |
| SIRT1 | p = 0.9074 | Mean = 1.21,  SD = 0.21 | Mean = 1.162,  SD = 0.13 | Mean = 1.11,  SD = 0.55 |
| α-KLOTHO | p = 0.5419 | Mean = 1.33,  SD = 0.415 | Mean = 1.15,  SD = 0.239, p = 0.445 | Mean = 1.07,  SD = 0.438, p = 0.324 |
| P53 | p = 0.9246 | Mean = 1.11,  SD = 0.20 | Mean = 1.19,  SD = 0.29 | Mean = 1.18,  SD = 0.67 |
| P16 | p = 0.3325 | Mean = 0.99,  SD = 0.286 | Mean = 1.64,  SD = 0.872, p = 0.3387 | Mean = 1.5,  SD = 0.782, p = 0.2023 |
| TERT | p = 0.392 | Mean = 1.4,  SD = 0.224 | Mean = 1.12,  SD = 0.502, p = 0.167 | Mean = 1.01,  SD = 0.566, p = 0.298 |
| IL6 | p = 0.2504 | Mean = 0.85,  SD = 0.15 | Mean = 1.03,  SD = 0.37 | Mean = 1.14,  SD = 0.3 |
| IL8 | p = 0.7529 | Mean = 1.14,  SD = 0.24 | Mean = 1.35,  SD = 0.27 | Mean = 1.28,  SD = 0.63 |
| Mitochondrial DNA content | p = 0.0129 | Mean = 0.721,  SD = 0.170 | Mean = 1.117,  SD = 0.398, p = 0.0407 | Mean = 1.035,  SD = 0.187, p = 0.00153 |
| Telomerase activity | p = 0.1765 | Mean = 0.00012 amole,  SD = 5.67E-05 amole | Mean = 0.00011 amole,  SD = 4.83E-05 amole | Mean = 0.0001 amole,  SD = 4.97E-05 amole |
| Telomere length | p = 0.9332 | Mean = 1.007,  SD = 0.26 | Mean = 1.216,  SD = 0.312 | Mean = 1.028,  SD = 0.364 |

**Table S2b: Statistical analysis for gene expression in the mice kidney tissue**

| **Kidney** | | | | |
| --- | --- | --- | --- | --- |
| **Genes analysed** | **Anova** | **Mice overexpressing LRP::FLAG** | **Control mice** | **Non-treated mice** |
| LRP/LR | p = 0.0111 | Mean = 1.85,  SD = 0.59 | Mean = 1.00,  SD = 0.36, p = 0.000839 | Mean = 1.13,  SD = 0.59, p = 0.0297 |
| c-MYC | p = 0.0082 | Mean = 1.73,  SD = 0.47 | Mean = 1.24,  SD = 0.35, p = 0.0573 | Mean = 1.01,  SD = 0.18, p = 0.0063 |
| MDM2 | p = 0.2588 | Mean = 1.29,  SD = 0.52 | Mean = 1.00,  SD = 0.45 | Mean = 0.93,  SD = 0.35 |
| SIRT1 | p = 0.0114 | Mean = 1.761,  SD = 0.794 | Mean = 1.023,  SD = 0.225, p = 0.02489 | Mean = 1.006,  SD = 0.369, p = 0.02479 |
| α-KLOTHO | p = 0.0221 | Mean = 1.86,  SD = 0.555 | Mean = 1.24,  SD = 0.470, p = 0.0369 | Mean = 1.12,  SD = 0.518, p = 0.0153 |
| P53 | p = 0.0363 | Mean = 1.87,  SD = 0.609 | Mean = 1.29,  SD = 0.312, p = 0.0402 | Mean = 1.18,  SD = 0.564, p = 0.0302 |
| P16 | p = 0.1079 | Mean = 0.793,  SD = 0.096 | Mean = 1.189,  SD = 0.489, p = 0.105 | Mean = 1.011,  SD = 0.171, p = 0.0268 |
| TERT | p = 0.0351 | Mean = 2.16,  SD = 0.95 | Mean = 1.23,  SD = 0.573, p = 0.0725 | Mean = 1.14,  SD = 0.572, p = 0.0483 |
| IL6 | p = 0.0664 | Mean = 0.63,  SD = 0.24 | Mean = 0.97,  SD = 0.2, p = 0.0175 | Mean = 1.16,  SD = 0.66, p = 0.0366 |
| IL8 | p = 0.3409 | Mean = 0.81,  SD = 0.53 | Mean = 1.05,  SD = 0.68 | Mean = 1.37,  SD = 0.79 |
| Mitochondrial DNA content | p = 0.0009 | Mean = 0.776,  SD = 0.220 | Mean = 1.386,  SD = 0.369, p = 0.0036 | Mean = 1.057,  SD = 0.301, p = 0.0322 |
| Telomerase activity | p = 0.0037 | Mean = 0.0025 amole,  SD = 0.00075 amole | Mean = 0.0016 amole,  SD = 0.00033 amole, p = 0.0254 | Mean = 0.0013 amole,  SD = 0.00028 amole, p = 0.0072 |
| Telomere length | p = 0.0065 | Mean = 1.56,  SD = 0.167 | Mean = 1.02,  SD = 0.319, p = 0.0191 | Mean = 1.01,  SD = 0.470, p = 0.0337 |

**Table S2c: Statistical analysis for gene expression in the mice liver tissue**

| **Liver** | | | | |
| --- | --- | --- | --- | --- |
| **Genes analysed** | **Anova** | **Mice overexpressing LRP::FLAG** | **Control mice** | **Non-treated mice** |
| LRP/LR | p = 0.0079 | Mean = 1.435,  SD = 0.557 | Mean = 0.738,  SD = 0.114, p = 0.005389 | Mean = 0.932,  SD = 0.328, p = 0.012578 |
| c-MYC | p = 0.0252 | Mean = 1.41,  SD = 0.372 | Mean = 1.18,  SD = 0.505, p = 0.372 | Mean = 0.83,  SD = 0.186, p = 0.002851 |
| MDM2 | p = 0.2381 | Mean = 1.06,  SD = 0.33 | Mean = 0.78,  SD = 0.16 | Mean = 1.1,  SD = 0.48 |
| SIRT1 | p = 0.0038 | Mean = 1.35,  SD = 0.139 | Mean = 0.92,  SD = 0.186, p = 0.0000343 | Mean = 1.03,  SD = 0.288, p = 0.02264 |
| α-KLOTHO | NA | NA | NA | NA |
| P53 | p = 0.0202 | Mean = 1.38,  SD = 0.472 | Mean = 0.75,  SD = 0.184, p = 0.00648 | Mean = 0.937,  SD = 0.475, p = 0.0853 |
| P16 | p = 0.1031 | Mean = 0.66,  SD = 0.36 | Mean = 0.44,  SD = 0.2 | Mean = 0.94,  SD = 0.44 |
| TERT | p = 0.1031 | Mean = 1.64,  SD = 0.89 | Mean = 1.021,  SD = 0.352, p = 0.082 | Mean = 1.079,  SD = 0.433, p = 0.121 |
| IL6 | p = 0.6677 | Mean = 1.63,  SD = 1.24 | Mean = 1.29,  SD = 0.51 | Mean = 1.26,  SD = 0.39 |
| IL8 | p = 0.9921 | Mean = 1.14,  SD = 0.74 | Mean = 1.15,  SD = 0.78 | Mean = 1.01,  SD = 0.71 |
| Mitochondrial DNA content | p = 0.0132 | Mean = 0.65,  SD = 0.12 | Mean = 1.16,  SD = 0.408, p = 0.0274 | Mean = 1.06,  SD = 0.363, p = 0.0069 |
| Telomerase activity | p = 0.0177 | Mean = 0.0047 amole,  SD = 0.0019 amole | Mean = 0.0026 amole,  SD = 0.00068 amole,  p = 0.0399 | Mean = 0.0024 amole,  SD = 0.00096 amole,  p = 0.0319 |
| Telomere length | p = 0.0072 | Mean = 1.59,  SD = 0.514 | Mean = 0.93,  SD = 0.392, p = 0.0269 | Mean = 0.98,  SD = 0.376, p = 0.0266 |

**Table S3a: Statistical analysis for protein expression in the mice brain tissue**

| **Brain** | | | | |
| --- | --- | --- | --- | --- |
| **Proteins analysed** | **Anova** | **Mice overexpressing LRP::FLAG** | **Control mice** | **Non-treated mice** |
| LRP/LR | p = 0.0045 | Mean = 121.92%,  SD = 14.52% | Mean = 101.12%,  SD = 18.16%,  p = 0.0138 | Mean = 100%,  SD = 15.45%,  p = 0.0021 |
| c-Myc | p = 0.0009 | Mean = 185.98%,  SD = 74.01% | Mean = 123.4949%,  SD = 29.37%,  p = 0.000839 | Mean = 105.879%,  SD = 34.76%,  p = 0.000839 |
| MDM2 | p = 0.006 | Mean = 149.73%,  SD = 60.26% | Mean = 96.65%,  SD = 30.72%,  p = 0.0175 | Mean = 99.99%,  SD = 12.98%,  p = 0.0162 |
| pMDM2 | p = 0.6682 | Mean = 95.61%,  SD = 13.58% | Mean = 96.5%,  SD = 14.22% | Mean = 100%,  SD = 9.58% |
| SIRT1 | p = 1.922E-08 | Mean = 139.64%,  SD = 15.95% | Mean = 100.47%,  SD = 12.96%,  p = 6.099E-06 | Mean = 100%,  SD = 9.96%,  p = 7.584E-07 |
| α-klotho  (Full length) | p = 7.594E-06 | Mean = 239.98%,  SD = 76.65% | Mean = 92.23%,  SD = 74.95%,  p = 0.00034 | Mean = 99.99%,  SD = 44.73%,  p = 0.000036 |
| α-klotho  (Secreted isoform) | p = 0.0002 | Mean = 121.23%,  SD = 18.31% | Mean = 83.50%,  SD = 21.78%,  p = 0.0008 | Mean = 97.63%,  SD = 12.69%,  p = 0.0025 |
| p53 | p = 0.0038 | Mean = 183.70%,  SD = 58.70% | Mean = 111.89%,  SD = 42.88%,  p = 0.0072 | Mean = 125.10%,  SD = 34.81%,  p = 0.0148 |
| p16 | p = 0.0134 | Mean = 65.11%,  SD = 20.52% | Mean = 89.71%,  SD = 35.04%,  p = 0.163 | Mean = 103.79%,  SD = 26.23%,  p = 0.00057 |
| γH2AX | p = 0.000016 | Mean = 55.73%,  SD = 17.24% | Mean = 88.88%,  SD = 26.59%,  p = 0.0161 | Mean = 100%,  SD = 13.86%,  p = 7.4688E-07 |
| TERT | p = 0.0046 | Mean = 153.36%,  SD = 62.44% | Mean = 82.14%,  SD = 39.93%,  p = 0.0118 | Mean = 100%,  SD = 27.77%,  p = 0.0138 |
| pTERT | p = 0.0003 | Mean = 148.53%,  SD = 35.58% | Mean = 117.20%,  SD = 22.80%,  p = 0.0122 | Mean = 100%,  SD = 18.35%,  p = 0.00064 |

**Table S3b: Statistical analysis for protein expression in the mice kidney tissue**

| **Kidney** | | | | |
| --- | --- | --- | --- | --- |
| **Proteins analysed** | **Anova** | **Mice overexpressing LRP::FLAG** | **Control mice** | **Non-treated mice** |
| LRP/LR | p = 0.000062 | Mean = 128.24%,  SD = 25.97% | Mean = 82.62%,  SD = 16.30%,  p = 0.000722 | Mean = 91.51%,  SD = 16.31%,  p = 0.000136 |

**Table S3c: Statistical analysis for protein expression in the mice liver tissue**

| **Liver** | | | | |
| --- | --- | --- | --- | --- |
| **Proteins analysed** | **Anova** | **Mice overexpressing LRP::FLAG** | **Control mice** | **Non-treated mice** |
| LRP/LR | p = 0.0003 | Mean = 144.91%,  SD = 33.06% | Mean = 91.35%,  SD = 18.42%  p = 0.0008 | Mean = 100%,  SD = 22.67%,  p = 0.0031 |
| c-Myc | p = 0.002 | Mean = 140.15%,  SD = 41.12% | Mean = 89.51%,  SD = 23.64%,  p = 0.003224 | Mean = 99.99%,  SD = 24.67%,  p = 0.012578 |
| MDM2 | p = 0.073 | Mean = 92.55%,  SD = 27.11% | Mean = 85.64%,  SD = 35.24% | Mean = 97.13%,  SD = 19.11% |
| pMDM2 | p = 0.1715 | Mean = 92.99%,  SD = 12.82% | Mean = 107.24%,  SD = 27.09% | Mean = 100%,  SD = 8.23% |
| SIRT1 | p = 0.0002 | Mean = 260.22%,  SD = 119.87% | Mean = 140.35%,  SD = 48.48%,  p = 0.0092 | Mean = 100.42%,  SD = 31.59%,  p = 0.0012 |
| α-klotho  (Full length) | NA | NA | NA | NA |
| α-klotho  (Secreted isoform) | p = 0.0062 | Mean = 157.22%,  SD = 71.38% | Mean = 94.04%,  SD = 26.49%,  p = 0.0174 | Mean = 100%,  SD = 21.85%,  p = 0.0258 |
| p53 | p = 0.0045 | Mean = 135.71%,  SD = 30.03% | Mean = 95.80%,  SD = 23.09%,  p = 0.00123 | Mean = 108.55%,  SD = 29.71%,  p = 0.0447 |
| p16 | p = 0.0141 | Mean = 38.71%,  SD = 30.70% | Mean = 79.81%,  SD = 21.85%,  p = 0.0045 | Mean = 99.47%,  SD = 62.55%,  p = 0.0162 |
| γH2AX | p = 0.000012 | Mean = 52.86%,  SD = 14.02% | Mean = 95.20%,  SD = 26.69%,  p = 0.00112 | Mean = 100.18%,  SD = 23.71%,  p = 0.0000129 |
| TERT | p = 0.0071 | Mean = 144.70%,  SD = 45.55% | Mean = 94.87%,  SD = 22.91%,  p = 0.0094 | Mean = 100%,  SD = 28.11%,  p = 0.0185 |
| pTERT | p = 0.002 | Mean = 135.15%,  SD = 42.60% | Mean = 86.33%,  SD = 12.96%,  p = 0.0023 | Mean = 95.96%,  SD = 24.50%,  p = 0.0138 |

**Table S4: List of primers used for gene expression, mitochondrial DNA content, telomerase activity and telomere length analysis**

| **Gene Primers** | | | | |
| --- | --- | --- | --- | --- |
| **Name** | **Mouse Primers (5' - 3')** | | **Annealing Temp (°C)** | **Reference** |
| α-klotho | Forward | GATGGCAGAGAAATCAACACAGT | 60 | Sato et al., 2015 |
|  | Reverse | ACTACGTTCAAGTGGACACTACT |  |  |
| cMyc | Forward | TGAGGAGACACCGCCCAC | 55 | Magri et al., 2014 |
|  | Reverse | CAACATCGATTTCTTCCTCATCTTC |  |  |
| GAPDH | Forward | CAACTACATGGTCTACATGTTC | 55 | Sato et al., 2015 |
|  | Reverse | CGCCAGTAGACTCCACGAC |  |  |
| IL-6 | Forward | CCAGAGATACAAAGAAATGATGG | 58 | Li et al., 2010 |
|  | Reverse | ACTCCAGAAGACCAGAGGAAAT |  |  |
| IL8 | Forward | TGTCCCCAAGTAACGGAGAAA | 56 | Thangavel et al., 2014 |
|  | Reverse | TGTCAGAAGCCAGCGTTCAC |  |  |
| LRP | Forward | GGT TGG GAT TGC CAG ATG GA | 60 | - |
|  | Reverse | AAG TGC TAT TCA CAG GCC CC |  |  |
| MDM2 | Forward | ATTGCCTGGATCAGGATTCAGTT | 58 | Alder et al., 2015 |
|  | Reverse | ACCTCATCATCCTCATCTGAGA |  |  |
| p16 | Forward | CGGTCGTACCCCGATTCAG | 60 | Diekman et al., 2018 |
|  | Reverse | GCACCGTAGTTGAGCAGAAGAG |  |  |
| p53 | Forward | CTGTGCAGTTGTGGGTCAGC | 60 | Kim et al., 2012 |
|  | Reverse | ACCTCCGTCATGTGCTGTGA |  |  |
| RPLPO | Forward | GGACCCGAGAAGACCTCCTT | 60 | Lin et al., 2013 |
|  | Reverse | GCACATCACTCAGAATTTCAATGG |  |  |
| SIRT1 | Forward | TGTGAAGTT ACTGCAGGAGTGTAAA | 56 | Bai et al., 2011 |
|  | Reverse | GCATAGATACCGTCTCTTGATCTGAA |  |  |
| TERT | Forward | TGCTACTGTTGAGCCGAGTG | 56 | - |
|  | Reverse | GCCTGTAACTAGCGGACACA |  |  |
| Mt DNA content primers | | | | |
| mtDNA specific (murine) | Forward | CCGCAAGGGAAAGATGAAAGAC | 60 | Bai et al., 2011 |
|  | Reverse | TCGTTTGGTTTCGGGGTTTC |  |  |
| nuclear specific (murine) | Forward | GCCAGCCTCTCCTGATTT TAGTGT | 60 | Bai et al., 2011 |
|  | Reverse | GGGAACACAAAAGACCTC TTCTGG |  |  |
| Telomere length related primers | | | | |
| Tel 1 | Forward | TCCCGACTATCCCTATCCCTATCCCTATCCCTATCCCTA | 60 | Cawthon, 2002 |
| Tel 2 | Reverse | GGTTTTTTGAGGGTGAGGGTGAGGGGTGAGGGTGAGGGT |  |  |
| 36B4 | Forward | ACTGGTCTAGGACCCGAGAAG | 60 | O'Callaghan, and Fenech, 2011 |
|  | Reverse | TCAATGGTGCCTCTGGAGATT |  |  |
| Telomerase activity primers | | | | |
| TS | Forward | AATCCGTCGAGCAGAGTT | 58 | Kim et al., 1997 |
| ACX | Reverse | GCGCGGCTTACCCTTACCCTTACCCTA |  |  |

**Table S5: Antibodies and dilutions used for western blotting analysis**

| **Target Protein** | **Antibody name** | **Dilution Factor** |
| --- | --- | --- |
| **Primary antibody** | | |
| β-actin | Anti-β-actin-peroxidase (Sigma A3854) | 1:10000 |
| γH2AX | Anti-PHOSPHO-H2AFX (SAB4300213) | 1:1000 |
| c-Myc | Anti-MYC (AB-58) (Rabbit) (Sigma-Aldrich: SAB4300318) | 1:1000 |
| Klotho | Anti-KL (Rabbit) (Sigma-Aldrich: SAB2101270-50UG) | 1:1000 |
| LRP/LR | Anti-LRP/LR IgG-iS18 (Affimed) | 1:6500 |
| MDM2 | Anti-MDM2 (Rabbit) (Sigma-Aldrich: SAB4501849) | 1:1000 |
| pMDM2 | Anti-phospho-MDM2 (pSer166) (Rabbit) (Sigma-Aldrich: SAB4503937) | 1:1000 |
| p16 | Anti-p16 INK, N-Terminal (Rabbit) (Sigma-Aldrich: SAB4500072) | 1:500 |
| p53 | Anti-p53, C-Terminal (Rabbit) (Sigma-Aldrich: SAB4503015) | 1:500 |
| SIRT1 | Anti-SIRT1 (Rabbit) (Sigma-Aldrich: AV32386-100UL) | 1:1000 |
| TERT | Anti-TERT/ Telomerase (Rabbit) (Lifespan Biosciences: LS-C312849-100 | 1:1000 |
| pTERT | Anti-phospho-Telomerase (pSer824) (Rabbit) (Sigma-Aldrich: SAB4504295) | 1:1000 |
| **Secondary antibody** | | |
| Anti-human IgG-HRP (Abcam 6858) | | 1:6500 |
| Anti-rabbit IgG-HRP (Cell signalling Technologies: 7074S) | | 1:2500 |
